# Poly(ethylene furanoate) (PEF): Advances in Synthesis, Properties, Recycling, Applications, and Future Challenges

**DOI:** 10.1021/acspolymersau.5c00189

**Published:** 2026-03-24

**Authors:** Purabi Bhagabati, Laura Cahill, Urbain N. Ndagano, Graham Reid, Meabh Kennedy, Ciara Tobin, Emma Nolan, Dylan Doherty, Susan M. Kelleher

**Affiliations:** School of Chemical Sciences, Dublin City University, D09 E432 Dublin, Ireland

**Keywords:** poly(ethylene furanoate), PEF, biobased polyesters, 2,5-furandicarboxylic acid, FDCA, sustainable packaging, barrier packaging, circular economy

## Abstract

The increasing concern over plastic pollution mostly due to extensive use of petroleum-based poly­(ethylene terephthalate) (PET) has intensified the search for sustainable biobased alternatives. Poly­(ethylene furanoate) (PEF), a fully biobased polyester derived from renewable feedstocks, has emerged as one of the most promising candidates. With superior gas-barrier performance, strong mechanical properties, and the potential for lower carbon emissions, PEF has attracted significant attention as a viable material to replace PET in several applications. This perspective presents an up-to-date and comprehensive overview of scientific and technological developments in PEF, tracing progress from its early discovery to its current industrial relevance. Particular emphasis is placed on the chemistry of PEF synthesis, including recent advances in greener pathways, as well as the structure–property relationships that refer to its superior thermal, mechanical, and barrier properties compared to PET. Performance characteristics arising from chemical structure and molecular modifications are also discussed. The review further examines the present landscape of PEF recycling, covering mechanical, chemical, and emerging enzymatic methods and integrates findings from recent life cycle assessment and techno-economic analysis studies to evaluate its environmental and economic viability. Industrial applications and associated challenges are explored, with a focus on packaging, where PEF’s barrier and mechanical advantages offer clear benefits over PET and multilayer systems. The paper concludes by outlining key research gaps that must be addressed to enable scalable, circular, and industrial deployment of the PEF.

## Graphical abstract

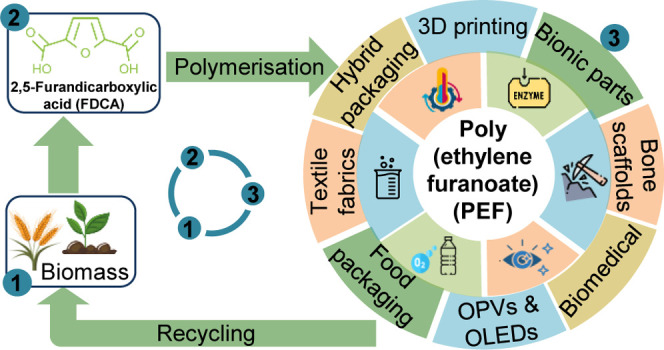

## 1 Introduction

Plastics are an integral part of modern society and play vital roles in various aspects of our daily lives. The high durability and low maintenance requirements of numerous plastic products used in homes and businesses, both indoors and outdoors, enhance their reusability, ultimately reducing associated costs. They play a crucial role in preventing food waste by enabling efficient distribution worldwide without compromising food quality. Plastic packaging helps preserve food freshness for extended periods, making it possible to transport food over long distances, which would otherwise be difficult to achieve. Plastic packaging also stands out from other packaging materials like glass, cardboard, and wood by enabling the transportation of larger quantities of goods with reduced fuel consumption, leading to significantly lower CO_2_ emissions. However, plastic waste accumulates in the environment when its rate of entry surpasses the capacity of natural or human-led cleanup processes, referred to as “plastic pollution”. Plastic pollution is deemed “a poorly reversible pollutant” due to the extremely slow natural mineralization process.^1^ The sharp rise in the global population and associated urbanization and economic development directly influences the increasing demand for plastic production. The rising consumer demand for plastic inevitably leads to a corresponding increase in plastic waste, which must be managed properly to prevent or minimize plastic pollution. As per a predictive modeling report by “Organisation for Economic Co-operation and Development (OECD)”, under the current scenario, global plastic waste is projected to triple by 2060, with half of it ending up in landfill with less than a fifth being recycled. In the report, the plastic leakage into the environment is projected to reach 44 million metric tons a year.^2^ This plastic waste raises multiple concerns due to their impact, not only to human health but also on Earth’s entire ecosystem.^3^ Previous studies have shown that plastic debris in the form of micro and nano plastic can harm the biota of aquatic and terrestrial ecosystems both physically and chemically.^4–7^ Moreover, there are significant effects on our climate as a result of plastics and plastic waste.

While only 6% of the world’s oil is used to make plastics, nearly 99% of plastics production is reliant on fossil feedstocks.^8^ The production of plastics from fossil feedstock is highly carbon-intensive, generating an estimated from 1.96 to 2.24 Gigatons of CO_2_ emissions from 2015 to 2019.^9,10^ In other words, the existence of fossil-based plastic threatens the current human effort to keep global temperature rise below 1.5 °C.^8,11^ At present, fossil-based plastic is responsible for 4.5% of global greenhouse gas emissions, which may not seem entirely threatening initially.^12^ However, with the continuation of the current global trend in plastic demand and production, the estimated greenhouse gas (GHG) emission from plastics would reach 15% of the global carbon budget by 2050.^13^ Interestingly, the plastic recycling processes come with their own share of GHG emissions as a result of the complicated process of collection, transportation, sorting, and treatment prior to its recycling. In a projection, modeling performed by the OECD estimates that global CO_2_ emissions from the plastics lifecycle will rise to 4.3 Gigatons by 2060 if current policies remain unchanged.^2^ In other words, these commodity plastics that are produced from fossil feedstocks leave a significant carbon footprint on the environment. The most significant source of plastic consumption is packaging, accounting for 40% of the total usage across all industries. As per reports, food packaging is the fastest growing and largest driver of global plastic packaging applications.^14,15^ Among different commodity plastics, poly­(ethylene terephthalate) (PET) holds the largest share of the market demand in the food packaging industry, followed by polyethylene (PE), poly­(vinyl chloride) (PVC), and polypropylene (PP).^15^ The convenience of PET with considerable flexibility in molding as required, high moisture barrier properties, and mechanical strength leads to its popularity as a material of choice. The growing demand for imported food products with substantial food miles, advancements in food product innovation, the rise of supermarkets, extended storage requirements, and increasing disposable household income are driving the need for PET production. In 2021 alone, the annual production of PET was over 55 million tons globally. While its properties have made it a preferred choice in food and beverage packaging, these same qualities also mean it can persist in the environment for centuries, with PET packaging accounting for about 12% of global solid waste.^16^

Even in recycling, the CO_2_ emission associated with production of 1 kg of virgin or mechanically recycled petroleum-based PET pellets is higher than widely used other commodity plastic PE and PP (see Table 1).^17^ Given the higher GHG emissions associated with PET in comparison to those of PE and PP, it has become evident that alternatives to PET need to be explored. These alternatives should not only match PET’s performance during service but also ensure lower GHG emissions throughout their entire lifecycle. Table 1 presents comparative data on the “Global Warming Potential (GWP)” of the commodity plastics derived from nonrenewable and renewable sources within the cradle-to-gate system boundary. Analysis shows that biobased counterparts of many conventional petroleum-based plastics have lower GWP (Table 1).

**Table 1:** Global Warming Potential (GWP) of Different Plastics from Cradle-to-Gate (Polymer Production from Raw Materials), kg CO_2_ eq/kg Polymer^17–21^

plastic type	GWP (kg CO_2_ e)/kg material
virgin PET granules	2.23
virgin Bio-PET granules	1.23
recycled PET	0.91
virgin HDPE granules	1.89
virgin Bio-PE granules	0.90
recycled HDPE	0.56
virgin PP granules	1.84
virgin Bio-PP granules	–0.06
recycled PP	0.53
virgin PVC granules	2.51
virgin PS granules	3.68
polyurethane (PUR) granules	5.70
virgin Bio-PUR granules	2.75
virgin poly(hydroxy alkanoates) (PHA)	0.12
virgin PLA granules	0.92
^a^starch-based PEF granules	1.46
^b^wheat-based PEF granules	0.99

a Assessment method GWP100a from Intergovernmental Panel on Climate Change (IPCC).

b Reference 20.

The low GWP of poly­(lactic acid) (PLA), one of the well-known biobased and biodegradable plastics available in the market, makes it an excellent choice for multiple single use packaging applications. However, PLA is not suitable to replace PET in many food and beverage packaging applications that need high gas barrier properties. Amorphous PET offers better transparency, toughness, and resistance to high temperatures; in contrast, PLA has limitations in these properties, making it a less desirable candidate for storage of cold and hot beverages that need long shelf life. PEF is a biobased, biodegradable plastic that has captured significant attention from both industries and the academic research community in recent years.^22^

### 1.1 Poly­(ethylene furanoate)

The PEF is a 100% biobased “Alipharomatic polyester” that shares structural similarities with PET (see Figure 1), the synthesis of which was first reported as early as 1946 via bulk polytransesterification under elevated temperatures (>200 °C) and high vacuum.^23,24^ However, the literature on PEF remained limited and fragmented until 2009 as PET dominated the field due to its combination of low production cost, ready availability, and excellent performance characteristics. A resurgence of interest occurred when Gandini et al. reported the synthesis of PEF through the transesterification of bis­(hydroxyethyl)-2,5-furandicarboxylate with ethylene glycol (EG) which is catalyzed by antimony­(III) oxide.^25^

**Figure 1:**
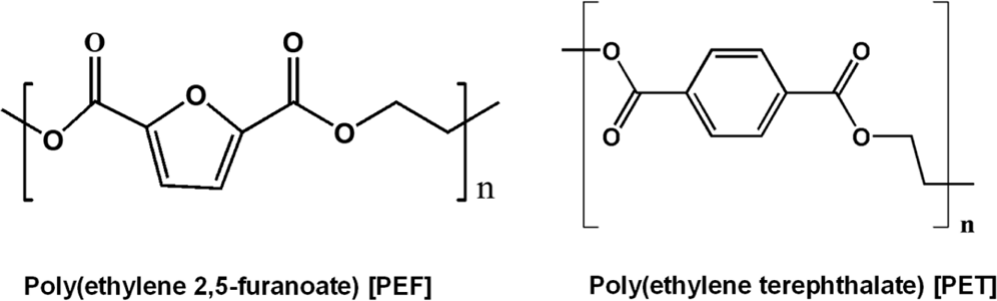
Chemical structure of the repeating units of PEF vs PET.

From then, PEF has attracted significant research interest due to its promising mechanical, thermal, and processing properties that closely resemble those of PET.^26^ In addition, the improved CO_2_, O_2_, and moisture barrier properties of PEF makes it a potential competitor to PET. All petroleum-based plastics receive a CO_2_ burden, whereas the biobased plastic deals with biogenic CO_2_. However, the released CO_2_ during the life cycle of plastics can be considered as emission regardless of the source of carbon (biogenic or petroleum based). Studies indicated that the GWP of PEF derived from wheat straw with a system boundary of cradle-to-gate is found to be at least 134–163% lower compared to its petroleum-based virgin PET.^20^ However, the CO_2_ emission benefit of biobased plastics over petroleum-based counterparts is generally realized during its end-of-life (EoL) option, i.e., recycling, composting, or incineration/energy recovery stage. An early Life Cycle Assessment (LCA) study with a cradle-to-grave system boundary was performed using ASPEN Plus simulation modeling to compare GHG emission of corn starch-based PEF with fossil-based PET. The study found that replacing PET with PEF could reduce GHG emissions by 45% to 55%, considering biogenic CO_2_ under the closed-loop carbon neutrality concept.^27^ Detailed discussion on PEF’s contribution toward GHG emission and nonrenewable energy use (NREU) in comparison to other petroleum as well as biobased plastics will be discussed in the subsequent section.

### 1.2 Current State of Research on PEF

PEF is the most promising biobased alternative to traditional petroleum-based PET for barrier packaging applications. Other than superior mechanical, thermal, and processability properties, neat PEF exhibits 11 times and 19 times better O_2_ and CO_2_ barrier properties, which can significantly extend the shelf life of carbonated beverages.^28,29^ The higher moisture barrier property of PEF makes it an ideal candidate for packaging moisture sensitive food products.^30^ The high melt strength and tensile properties of neat PEF may enable thinner packaging with the same durability, minimizing material use and providing a cost advantage. The higher glass transition temperature, melting point, and overall thermal stability of PEF make it ideal for holding hot beverages at 90 °C, offering a clear preference over PET. Tailoring the synthesis technique offers the flexibility to adopt the copolymerization with other moiety to alter the necessary properties of final products. Biobased and biodegradable monomers, such as lactides and lactones, can commonly be used to enhance PEF’s properties through chemical copolymerization. Physical blending of PEF with other polymers like PLA, poly­(butylene succinate) (PBS), and PET can help achieve a balance of desired properties suitable for specific applications. Similarly, inclusion of heterogeneous fillers in nano- or micro scale can enhance the property of resulting PEF composites. In other words, blending and copolymerization can enhance PEF’s properties to meet specific application requirements, details of which are covered in subsequent sections. The high gas barrier property of PEF may also allow it to compete with commercially available passive barrier materials such as ethylene vinyl alcohol (EVOH) in multilayer packaging. In current multilayer barrier packaging, complete separation of the barrier materials from the base polymer is a challenge. Due to the structural similarity in the backbones of PEF and PET, existing commercial PET recycling facilities can accommodate the mechanical and chemical recycling of PEF. One study indicates that presence of 2 wt % PEF in the PET waste stream (maximum allowed market penetration) does not interfere the overall recycling process or the recyclate.^31^ Another study suggests a safe limit of 5 wt % of PEF in PET showing performance remained unchanged.^32^ Therefore, PEF does not need to be separated from the PET waste stream if the level of contamination remains within the permissible limits. However, further research is needed to explore the sorting and recycling potential of PEF alongside PET and other similar polyesters like PLA.

### 1.3 Research Gap

Currently, the most common technique for the synthesis of PEF is melt polycondensation of 2,5-furandicarboxylic acid (FDCA) and ethylene glycol (EG) which is mostly followed by solid state polymerization (SSP). The reaction needs high temperature, long reaction time, and a strong vacuum system for efficient removal of water or other byproduct in order to shift the reaction equilibrium toward the product side. All of these factors contribute to the high energy demand of the polycondensation process. Also, high-temperature reaction conditions often degrade the product resulting in inconsistent molecular weight of PEF. The formation of byproduct reduces the final PEF quality and leads to coloration of the product.^33^ Ring-opening polymerization (ROP) is an alternative approach for the synthesis of PEF that offers several advantages over conventional techniques. Unlike polycondensation and SSP, it operates at milder temperatures, requires shorter reaction times, and eliminates the need for precise vacuum control systems. All of these factors collectively reduce energy demand for PEF production while enabling tight control over the molecular weight of PEF without causing thermal degradation or product discoloration. Absence of reaction byproducts in the ROP technique ensures the purity of PEF and makes it suitable for food contact applications. Despite the numerous advantages of the ROP technique for PEF synthesis, its industrial-scale implementation is challenging due to limited data in the literature.

### 1.4 Scope and Objective

Over the past decade, PEF has attracted significant research attention as a promising biobased alternative to petroleum-derived PET, largely motivated by the pressing global imperative to reduce dependence on petroleum-derived plastics and mitigate CO_2_ emissions. This perspective provides a comprehensive and structured narrative of the scientific advancements in PEF, considering its development from initial discovery to the most recent advancements. In addition to the coverage of scientific advancements in PEF, this review aims to identify and critically examine the key challenges and opportunities surrounding various aspects of PEF for a successful market integration such as the need for a coordinated supply chain, best practices for PEF synthesis and subsequent scalable production, modifications like copolymers, blends, and composites, processing, and adaptability with the existing PET-processing infrastructure. The review also addresses the infrastructure requirements for effective sorting and recycling, which is essential for the development of a sustainable end-of-life of PEF. Life cycle assessment (LCA) and techno-economic analysis (TEA) studies are reviewed to evaluate the sustainability and commercial feasibility of PEF. Current and potential applications of PEF, challenges associated with its translation from laboratory-scale research to industrial-scale implementation, and corresponding recommendations are also discussed. Literature focusing on the production or synthesis of FDCA is considered to be beyond the scope of this review.

### 1.5 Methodology

An appropriate search strategy was employed, with particular attention to thematic coherence and technical accuracy through the careful selection and inclusion of relevant literature. Comprehensive coverage of the relevant literature was ensured by sourcing publications from 1972 to mid-2025. The literature search was conducted and updated between January and June 2025 across Web of Science, SciFinder, and Google Scholar, utilizing the specific keywords: “poly­(ethylene furanoate)”, “poly­(ethylene furandicarboxylate)”, “PEF polymer”, and “poly­(2,5-furan dicarboxylate)”. To ensure wide coverage while maintaining specificity, searches with broader keywords such as “2,5-furandicarboxylic acid (FDCA)”, “furan-based polyesters/copolyesters”, “FDCA-polyesters”, and “furanic-aliphatic polyesters” were subsequently refined using the aforementioned specific keywords. Further filtering was accomplished by incorporating additional terms pertinent to the thematic focus of this review, including synthesis, melt polycondensation, solid-state polymerization (SSP), ring-opening polymerization, recycling, properties, copolymer, blends and composites, life cycle assessment (LCA), and techno-economic analysis (TEA). Literature not published in English and studies not specifically focused on PEF polymers were excluded from this review. A total of 214 relevant publications were identified, comprising 161 original research articles, 36 review papers, and 12 classified under other categories such as communications, perspectives, and analytical notes. Figure 2 provides an overview of the chronological distribution and thematic categorization of publications related to PEF. As shown in Figure 2A, research activity on PEF began to grow notably from 2014, which coincides with the rising interest in biobased alternatives to PET. The first review article on PEF was published in 2015, followed by a steady stream of review publications in subsequent years. Figure 2B highlights that a majority of studies have focused on PEF synthesis and properties. Research on PEF recycling has seen a marked increase since 2021, which indicates a shift toward end-of-life considerations. Interest in LCA, TEA, and application-driven research has also been gaining momentum since around 2017 in alignment with efforts to evaluate the sustainability of PEF-based products.

**Figure 2:**
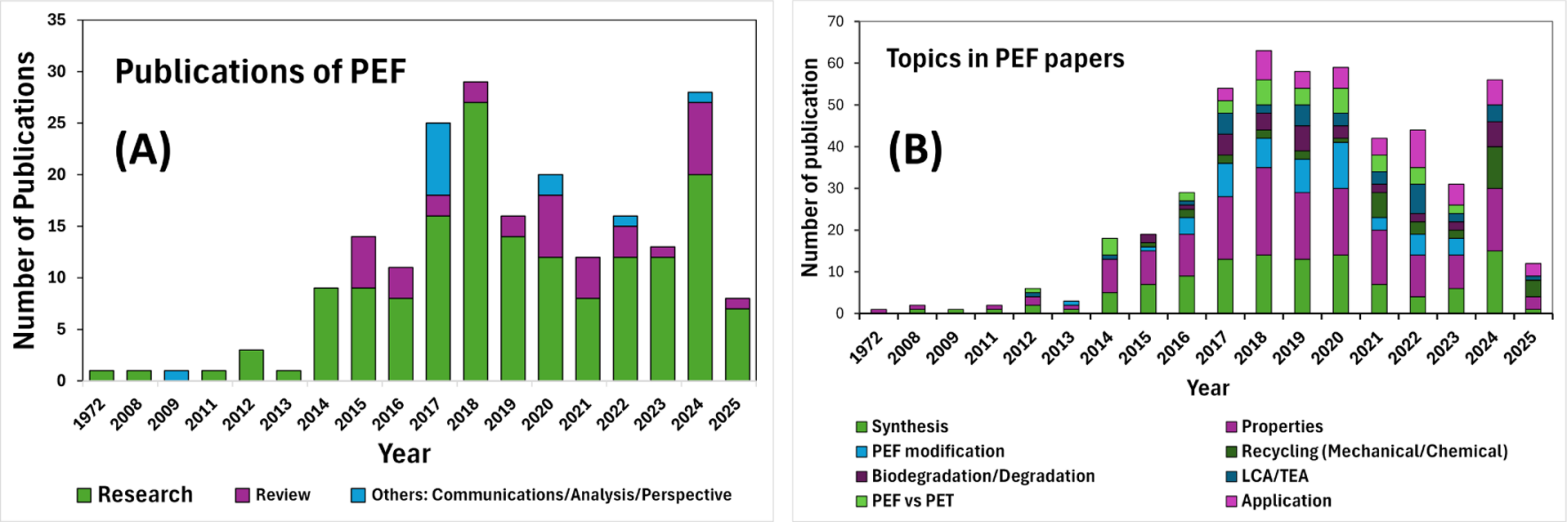
(A) Chronological distribution of published articles related to the PEF polymer. (B) Categorization of publications based on research areas: synthesis, material properties, chemical and physical modifications, recycling, biodegradation/degradation, LCA/TEA, comparative studies between PEF vs PET, and application development (data compiled according to the review methodology).

## 2 PEF Synthesis

### 2.1 PEF Raw Materials

FDCA and biobased EG are the major building blocks of PEF, and both are derived from biomass. EG is a commonly used material in chemical industries as an antifreeze, a coolant, a plasticizer of rigid plastics, a precursor of polyesters, and a heat transfer liquid in chemical engineering areas.^34^ FDCA is produced by catalytic conversion of biobased hydroxymethylfurfural (HMF). Currently, the raw material for HMF is edible biomass like sucrose from sugar cane and sugar beet which is converted into glucose or fructose.^35^ Utilizing nonedible polymeric biomass, such as lignocellulose (e.g., sawdust), and starch-based food waste (e.g., potatoes, wheat, rice) for HMF production provides the advantage of avoiding competition with food sources.^36^ Given the limited practicality of using edible biomass like sugars, lignocellulosic biomass is considered the most promising feedstock for HMF synthesis.^37,38^ This section provides a detailed overview and updated research on the synthesis of PEF, including a comparative analysis of the process’s technical aspects, advantages, and limitations along with implications based on various influencing factors.

The synthesis routes of PEF closely resemble those of PET, both involving melt polycondensation reactions through transesterification between dicarboxylic acids or esters with diols. While terephthalic acid (TA) is used for PET and FDCA is used for PEF synthesis (see Figure 3).

**Figure 3:**
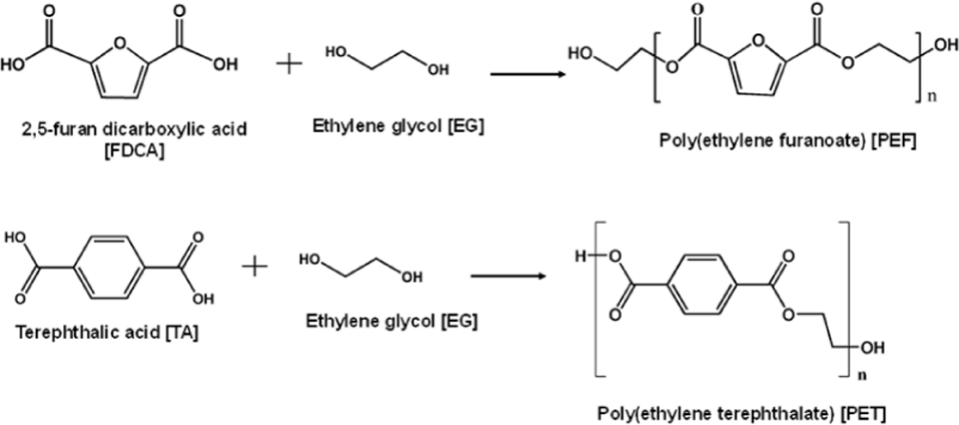
A simplistic schematic reaction for the synthesis of PEF and PET.

A common limitation of the melt polycondensation reaction is its inability to produce high-molecular-weight PEF, even under precise and rigorous control of reaction parameters. However, achieving high-molecular weight is critical for major applications of PEF including injection blow molding for bottles, fiber spinning for textiles, and uniaxial stretching for extrusion-cast films. To overcome this limitation, an additional postpolymerization step known as solid-state polymerization (SSP) is employed. The SSP not only extends the polymer chains, which is necessary for the intended applications of PEF but also enhances its thermal and mechanical properties. Each of the PEF synthesis methods is discussed in the following section along with the focus on its merits and limitations to enable critical comparison and guide for process optimization.

### 2.2 Melt Polycondensation

Melt polycondensation is the most common process for the synthesis of PEF that involves two key steps. Esterification or transesterification was followed by polycondensation under high vacuum and temperature. An additional solid-state polymerization (SSP) step is required to increase the molecular weight of the final PEF product. In the first step, FDCA undergoes an esterification reaction with EG under an inert atmosphere, releasing water as a byproduct. Gandini et al. reported literature on the synthesis of PEF using the polytransesterification reaction between FDCA and EG, with low-molecular weight and with high reaction time.^25^ Alternatively, when using dimethyl 2,5-furandicarboxylate (DMFDC), a diester monomer, the reaction proceeds via transesterification with EG under inert gas, producing bis­(hydroxyethyl) 2,5-furan dicarboxylate (BHFDC) as an intermediate component and methanol as a byproduct. The intermediate BHFDC undergoes a polycondensation reaction at elevated temperature to produce PEF. During the polycondensation reaction, a high vacuum is applied to remove the byproduct methanol from the reaction mixture. This not only minimizes the risk of polymer oxidation but also drives the reaction equilibrium toward polymer formation, resulting in higher-molecular weight. A schematic of the complete reaction is presented in Figure 4. In both routes, low-molecular-weight PEF, commonly referred to as PEF oligomers, is initially formed. The second step is the polycondensation reaction, which is carried out at elevated temperature and high vacuum key to remove the byproduct of the condensation reaction. The molecular weight and overall yield of the resulting PEF are highly influenced by the efficient removal of byproducts, which is a challenging task due to the reversible nature of the reactions. Consequently, the PEF obtained upon polycondensation reaction typically exhibit insufficient chain length for practical applications and require further polymerization. The SSP step is continued at high temperature and vacuum conditions, which promotes an increase in the degree of polymerization and facilitates the effective removal of the methanol byproduct. Several studies, have highlighted the application and industrial relevance of SSP in the production of PEF suitable for intended applications.^39–41^ SSP is not only an important method for increasing the molecular weight and intrinsic viscosity of PEF but also highly efficient at large-scale industrial manufacturing, thereby supporting the commercial viability of PEF. Table 2 below presents the detailed literature references on PEF synthesis via a melt polycondensation process with SSP as an optional step. The table focuses on the effects of monomers, reaction temperature, reaction time, and catalyst type as reported across multiple studies conducted over time in the field of PEF synthesis.

**Figure 4:**
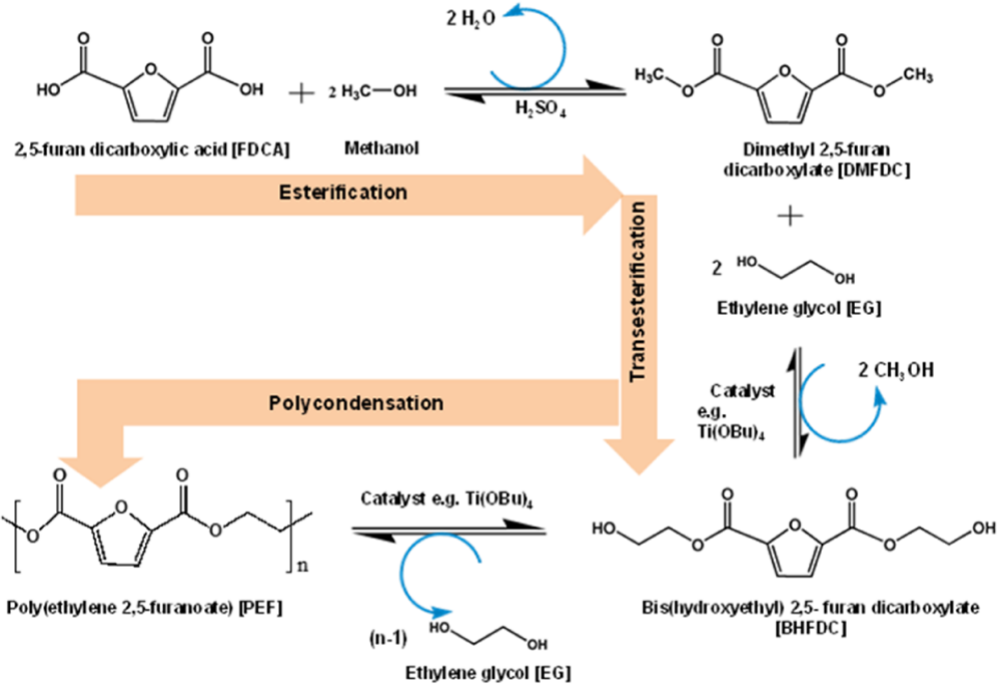
Synthesis of PEF via the polycondensation reaction.

**Table 2:** Literature on Melt Polycondensation Process for Synthesis of High Molecular Weight PEF

author, year	reaction type	monomers	catalyst	*T* (°C)	time (h)	vacuum	IV (dL/g)	MW (g/mol)
Gandini et al., 2009 ^25^	poly transesterification	FDCA, EG	antimony trioxide Sb_2_O_3_	220	-	high, unspecified	-	-
Gomes et al., 2011 ^47^	poly transesterification	DMFDC, EG	antimony trioxide Sb_2_O_3_	240–250	-	high, unspecified	-	*M*_n_ = 22,400*
Jiang et al., 2011 ^48^	direct esterification	FDCA, EG, 1,3-Propanediol, 1,4-Propanediol, 1,6-hexanediol, 1,8-octanediol	titanium(IV) *n*-butoxide Ti(OBu)_4_	235–245	-	inert gas	-	10.53 × 10^4^
Ma et al., 2011 ^49^	step 1: direct esterification	FDCA, EG, 1,4-butylene glycol (BG)	titanium(IV) *n*-butoxide Ti(OBu)_4_	160	6	inert gas	-	-
	step 2: SSP	-	titanium(IV) *n*-butoxide Ti(OBu)_4_	200	1.5	-	0.47	25,973 **
Gruter et al., 2012 ^50^	polycondensation in film reactor	DMFDC, EG	titanium(IV) isopropoxide Ti(OiPr)_4_	150–210	5	-	-	-
	polycondensation in film reactor	DMFDC, EG	titanium(IV) isopropoxide Ti(OiPr)_4_	240	3	12,500***	-	-
Gubbels et al.,2012 ^51^	bulk polycondensation	FDCA, DMFDC, 2,3-butanediol	tin(IV) ethylhexanoate	220	4	reduced pressure	-	7000
Knoop et al., 2013 ^52^	SSP × 1 times	FDCA, EG, 1,3-propanediol, 1,4-propanediol	titanium(IV) isopropoxide Ti(OiPr)_4_	195	2	1 × 10^–2^ mbar	-	*M*_n_ = 83,000^a^
Zhu et al., 2013 ^53^	step 1: direct esterification	FDCA, 1,4-butanediol (BDO) (1:3)	titanium tetraisopropoxide, Ti(OiPr)_4_	175–200	2–4	inert gas blanket	-	-
	Step 2: SSP	FDCA, 1,4-butanediol (BDO) (1:3)	titanium tetraisopropoxide, Ti(OiPr)_4_	200	8	0.13 mbar		*M*_w_ = 65,000^b^
Papageorgiou et al., 2014 ^42^	step 1: transesterification	DMFDC, EG	tetrabutyl titanate (TBT)	170–190	5	-	-	-
	step 2: polycondensation	DMFDC, EG	tetrabutyl titanate (TBT)	220–235–250	2–2–2	0.05 mbar	0.45	*M*_n_ = 11,200
Tsanaktsis et al., 2015 ^54^	step 1: transesterification	DMFDC, bishydroxyalkylene-2,5-furan carboxylate	tetrabutyl titanate (TBT)	150–170	5	argon	-	-
	step 2: polycondensation	DMFDC, bishydroxyalkylene-2,5-furan carboxylate	tetrabutyl titanate (TBT)	210–230	2	0.05 mbar	0.43–0.50	*M*_n_ = 34,500–39,900
Yu et al., 2016 ^55^	esterification	FDCA, EG	monobutyltin oxide (MBO)	180	-	-	0.71	*M*_n_ = 4.74 × 10^4^***
	transesterification	DMFDC, EG	monobutyltin oxide (MBO)	180	-	-	0.68	*M*_n_ = 4.64 × 10^4^***
Terzopoulou et al., 2017 ^33^	step 1: transesterification	DMFDC, EG	titanium(iv) isopropoxide (TIS), tetrabutyl titanate (TBT)	160–190	4	argon	-	-
	step 2: polycondensation	-	titanium(iv) isopropoxide (TIS), tetrabutyl titanate (TBT)	230	2	0.05 mbar	0.31	*M*_n_ = 6000
Kasmi et al., 2018 ^56^	SSP × 2 times	FDCA, EG	tetrabutyl titanate (TBT)	205	6	0.03–0.04 mbar	1.02	*M*_n_ = 33,900
Gomes et al., 2018 ^57^	step 1: esterification	FDCA, EG	-	175	2	nitrogen	-	-
	step 2: transesterification		antimony(III) oxide Sb_2_O_3_	220	7	–1013.2 mbar	-	-
	step 3: SSP		-	220–260	6	vacuum	-	*M*_n_ = 1.8 × 10^4^
Chebbi et al., 2019 ^58^	step 1: transesterification	DMFDC, EG	antimony(III) oxide Sb_2_O_3_	160–190	4	nitrogen	-	-
	step 2: polycondensation	-	-	230	2	0.05 mbar	-	-
	step 3: SSP	-	-	205	5	0.03–0.04 mbar	0.50	15,800
Höhnemann et al., 2021 ^59^	step 1: transesterification	FDCA, MEG	titanium(IV) tetra(*n*-butoxide)	200	4–7	nitrogen	-	-
	step 2: polycondensation	-	-	260	-	10 mbar	-	-
	Step 3: SSP	-	-	160–180	24–72	<10 mbar	0.85	55,900
Papageorgiou et al., 2021 ^60^	step 1: transesterification	DMFDC, EG	tetrabutyl titanate (TBT)	150–160	5	nitrogen	-	-
	step 2: polycondensation	-	-	230	2	0.05 mbar	-	-
	step 3: SSP	-	-	190–205	11	0.03–0.04 mbar	0.73	-
Stanley et al., 2023 ^61^	step 1: transesterification	DMFDC, EG	antimony(III) oxide Sb_2_O_3_	170–200	1.5	nitrogen	-	-
	step 2: polycondensation	-	-	250–260	6	0.05 mbar	0.64	16,500

a Size exclusion chromatography; Intrinsic viscosity (IV); Number-average molecular weight (*M*_n_); Molecular weight (MW); * Mark–Houwink’s equation using Cannon–Ubbelohde viscometer at 25 °C in the mixed solvent of phenol/1,1,2,2-tetrachloroethane (3:2, w/w); *** GPC.

b (PDI = 2.8).

The two-step reaction process for PEF synthesis that comprises of transesterification followed by polycondensation using DMFDC and EG was first reported by Papageorgiou et al., 2014.^42^ This approach was shown to be more efficient in achieving a higher-molecular weight PEF with better control over key reaction parameters. Generally, in esterification/transesterification reactions, the molar ratio of diacid/diester to diol is maintained close to the equimolar levels. For example, in the synthesis of PET, the molar ratio of terephthalic acid to ethylene glycol (EG) is often kept within the range of 1:1 to 1:1.3.^43^ On the other hand, the synthesis of PEF usually involves significantly lower molar ratios of FDCA/DMFDC to EG, ranging 1:2 to 1:5, which is primarily due to the poor solubility of FDCA in EG. Higher EG ratio enhances the dissolution of furan-based monomers, thereby creating a more uniform reaction medium that promotes effective esterification/transesterification. Moreover, FDCA and DMFDC exhibit higher reactivity compared to terephthalic acid or dimethyl terephthalate, which is due to the electron-withdrawing nature of the furan ring.^32^ The rigid structure of the furan ring imparts high stiffness to the PEF polymer chain, which can lead to increased viscosity during polymerization. The excess of EG improves polymer chain mobility and reduces viscosity, thereby facilitating better mixing, which are important factors in achieving higher-molecular weights. Additionally, the use of excess EG helps drive the esterification/transesterification equilibrium toward product formation that enhances both yield and molecular weight.^44^ This pathway also enables the reaction to proceed at lower temperatures and shorter durations, which minimizes the risk of thermal degradation and discoloration of the final PEF product. Other critical factors influencing the physical properties of PEF include reaction temperature, reaction time, as well as the type and concentration of the catalyst used. In melt polycondensation processes, routes involving esterification of FDCA mostly require ∼30–50 °C higher temperatures and ∼1–2 h longer reaction times compared to those employing transesterification of DMFDC.^32^ This is due to the lower reactivity and poor solubility of FDCA, which demands a longer residence time in the reaction chamber to achieve the desired molecular weight. In contrast, DMFDC is more reactive dimethyl ester and enables the polycondensation reaction to proceed more efficiently at lower temperatures.^45^ Since furan rings are thermally sensitive and prone to discoloration at elevated temperatures, DMFDC is often preferred as the monomer in applications where the coloration of PEF is highly undesirable. In polyester chemistry, a notable side reaction during melt polycondensation is the formation of diethylene glycol (DEG) via etherification of EG, which is a phenomenon well-documented in PET synthesis.^46^ As in the case of PET, the presence of DEG in the polymer chain can significantly alter the physical, thermal, and mechanical properties of the final PEF. DEG formation is particularly sensitive to several variables, including the molar excess of EG, reaction temperature, and duration, as well as the type and concentration of the metal catalyst employed. Since an excess of EG is mostly required for efficient PEF synthesis, a careful consideration of reaction conditions becomes necessary.

It has been reported that a wide variety of metal catalysts such as antimony (Sb), titanium (Ti), zinc (Zn), tin (Sn), magnesium (Mg), hafnium (Hf), zirconium (Zr), and manganese (Mn), germanium (Ge) are effective in synthesizing PEF via the melt polycondensation reaction. As summarized in Table 2, antimony-based catalysts (e.g., Sb_2_O_3_ and [Sb­(CH_3_COO)_3_]) and titanium-based alkoxides like titanium­(IV) *n*-butoxide and titanium­(IV) isopropoxide have demonstrated superior performance in achieving high-molecular weight of PEF, which is attributed to their high catalytic activity. However, this catalytic efficiency often comes at the cost of polymer discoloration, primarily due to decarboxylation of FDCA and formation of colored byproducts as reported by Dong et al.^62^ Similarly, zirconium and tin-based catalysts, such as zirconium­(IV) isopropoxide, titanium (TiO_2_) nanowire,^63^ and dibutyltin­(IV) oxide,^64^ are reported to cause discoloration in PEF under high-temperature conditions.

### 2.3 Green” Synthesis

Though these metal catalysts perform well, the major limitation lies with metals becoming part of the final PEF product, which may show potential health and environmental risks.^22^ The inherently existing challenges in the melt polycondensation process for PEF synthesis include use of high temperature and vacuum for longer durations that leads to high energy consumption, FDCA degradation, and unwanted byproduct formation, leading to discoloration of the final PEF product. Hence, a demand for a sustainable process for PEF synthesis has arisen in relation to the growing concern about environmental aspects. Table 3 summarizes the reported studies on “green” synthesis of PEF including details on reaction parameters, conditions, and their impact on the resulting PEF molecular weight (MW). The environmental and health risks associated with the heavy metals catalysts can be addressed by using biocompatible metal catalysts like Zn, iron (Fe), Mg, and calcium (Ca) powders, offering greener alternative to the heavy metal catalysts. Out of all these metal catalysts, Zn metal powder-based catalysts offered satisfactory results with highest molecular weight (MW) and light color appearance.^65^ These catalysts do not need be removed from the final PEF polymer, and they can become part of the product without issue. Attempts have been made to find a greener alternative of heavy metal-based catalysts such as nonmetallic 8-diazabicyclo [5.4.0] undec-7-ene (DBU) as the efficient catalyst.^66^ A recent report by Jiang et al., 2020 studied the environmental impact of PEF made from CO_2_, focusing on FDCA production from captured CO_2_ and biowaste furfural.^67^ Since this is outside the scope of our review, it is not discussed here or included in Table 3, but the study is discussed in detail in Section 5 of this article. The subsequent discussion focuses on different ‘green’ routes for the synthesis of PEF.

**Table 3:** Literature on “Green” Synthesis of PEF with Key Process Parameters and Characteristics^a^

author, year	reaction type	reaction steps	monomers	catalyst and concentrations	pressure	*T* (°C)	time (h)	MW(*M*_n_)* (g/mol)	intrinsic viscosity
Wu et al., 2016 ^66^	MPC	step 1: transesterification	DMFDC/EG = 1:2.5	8-diazabicyclo [5.4.0]undec-7-ene (DBU)	N_2_, 1 bar	170–200	6–8	-	-
		step 2: polycondensation		0.1 mol % of DMFDCA	1 mbar	230–240	4.5		0.54 dL/g
Qu et al., 2019 ^69^	MPC	step 1: transesterification	FDCA/EG = 1:2.5	ILs: 1-alkyl-3-methylimidazolium tetrafluoroborate, [C_n_MIM]BF_4_	N_2_, 1 bar	200–240	-	-	-
		step 2: polycondensation	-	0.1 mol % of FDCA	30 mbar	240–260	6	4.19 × 10^4^	0.54 dL/g
Qu et al., 2021 ^65^	MPC	step 1: transesterification	DMFDC/EG = 1: 2.0	Zn powder, 0.1% based on DMFDCA (mol/mol)	N_2_, 1 mbar	170–190	4		
		step 2: polycondensation			20 mbar	230–240	5	4.8 × 10^4^	
Zhou et al., 2024 ^70^	MPC	step 1: esterification	FDCA/EG = 1:1.96	ILs: Acidic Metal-Based Ionic Liquid Catalyst [DA-2PS][SnCl_5_]_2_, triethylenediamine, PS = 1,3-propanesultone, concentration = 0.05 mol % of FDCA		220	-	-	-
		step 2: polycondensation	-			240	6.3	28,820 g/mol	0.68 dL/g
Jiang et al., 2015 ^74^	EP	step 1: transesterification	DMFDC/1,10-decanediol = 1:1 (1,10-DDO)	Novozym 435 (an immobilized form of Candida antarctica lipase B (CALB) on acrylic resin	N_2_, 1 bar	80	2	-	-
		step 2: polycondensation			2.67 mbar	80	72	23,700; 94% **	-
Comerford et al., 2020 ^75^	EP	step 1: transesterification	DMFDC/1,4-butanediol (1,4-BDO) = 1:1	Novozym 435 (an immobilized form of Candida antarctica lipase B (CALB) on acrylic resin	1000 mbar	50	6		-
		step 2: polycondensation	-		20 mbar	50	18	500 g/mol	-
		step 3: thermal treatment	-		vacuum	160	24	10,000 g/mol	-
Silvianti et al., 2024 ^76^	EP	one step polycondensation, solvent: *p*-cymene	DMFDC/1,10-decanediol = 1:1 (1,10-DDO)	Novozym 435 (an immobilized form of Candida antarctica lipase B (CALB) on acrylic resin	N_2_, 1 bar	90	72	***7000 g/mol	-
Morales-Huerta et al., 2016 ^80^	ROP	step 1: Depolymerization	2,5-Furandicarbonyl dichloride (FDCA-Cl_2_)	Diazabicyclo[2.2.2] octane (DABCO)	-	180	4 days	60%^	-
		step 2: ring-opening polymerization (ROP)	CyOEF	Tin(II) ethylhexanoate Sn(Oct)_2_		220–250	2	55,000 g/mol	-
Rosenboom et al., 2018 ^78,81^	ROP	step 1: depolymerization	DMFDC, EG	Dibutyltin oxide Bu_2_SnO		180–200	6–8	>99%^b^	-
		step 2: ring-opening polymerization (ROP)	CyOEF	1,1,6,6-tetra-*n*-butyl-1,6-distanna-2,5,7,10-tetraoxacyclodecane (Cyclic siloxane), 1-dodecanol and tetraglyme as plasticizer/initiator		220	<30 min	30,000 g/mol; >95%^b^	-

a MPC: Melt polycondensation; EP: Enzymatic polymerization; *GPC technique, PS standard; ** Isolation yield; *** Weight average molecular weight (*M*_w_).

b Yield percentage.

#### 2.3.1 Ionic Liquids as Catalysts

Ionic liquids (ILs) have started gaining attention for PEF synthesis due to their strong Brønsted/Lewis acidity and dual functionality as both catalyst and solvent for FDCA and EG under mild reaction condition. Their negligible vapor pressure, high thermal stability, and nonflammability reduce emissions and enhance process safety during melt polycondensation.^68^ In addition, the structural tunability of IL cations and anions allows great tunability of acidity, polarity, and coordination environment to match the requirements of the PEF synthesis.

The pursuit of greener alternatives for PEF synthesis led Qu et al. to explore the use of ILs as catalysts. Their study in 2019, demonstrated that ILs can serve as an environmentally friendly, efficient, and selective catalyst for producing high-molecular-weight PEF via melt polycondensation. In their study, a series of various dialkyl-substituted imidazole ILs were used as the catalyst out of which [C_2_MIM]­BF_4_ the catalyst with stronger electronegativity and proton-donating ability performs best in obtaining high-molecular-weight PEF.^69^ Later on 2023, Zhou et al. investigated a family of acidic metal-based functional ILs derived from dizwitterionic intermediate DA-2PS (triethylenediamine (DA), 1,3-propane sultone (PS)) and metal chlorides, [DA-2PS]­[XCly]_2_, as catalysts for the esterification of FDCA and EG. Among series of ILs, [DA-2PS]­[SnCl_5_]_2_ exhibited the best catalytic performance with minimum loading of 0.05 mol % based on FDCA. The obtained PEF with intrinsic viscosity of 0.68 dL·g^–1^ and a number-average molecular weight of 28,820 g·mol^–1^ indicates a great success for the use of ILs in PEF synthesis. This work demonstrates the possibility of broadening the use of ILs in PEF synthesis judiciously tuning their acidity, hydrophilicity, and biodegradability in order to balance catalytic performance with overall environmental sustainability.^70^ However, the high cost and the need for recyclability of ILs hinder their large-scale application in PEF synthesis. This challenge refers to the importance of designing low cost and more benign ILs and integrate robust recycling process like distillation, phase separation, or immobilization to make the IL-catalyzed PEF synthesis economically and environmentally viable at industry scale.

#### 2.3.2 Enzymatic Polymerization

Enzymes are biological catalysts that are inherently nontoxic, renewable, and recyclable. Their ability to facilitate reactions under mild conditions significantly reduces the environmental impact associated with conventional chemical processes. Despite these advantages, the enzymatic synthesis of PEF has not been studied greatly. However, Gkountela and Vouyiouka provided a concise review summarizing the recent understanding and offering guidance for the enzymatic production of PEF.^71^ The synthesis of low-molecular-weight polyester using lipase-catalyzed polymerization of diacids and diols was first published by Okumura et al. in 1984. In this study, the lipase was derived from *Aspergillus niger* NRRL 337.^72^ Building upon earlier work, Jiang et al. conducted a series of studies on the enzymatic polymerization of furan-based monomers to investigate the influence of various factors like diol type, diol chain length, and reaction conditions on the chemical structure and properties of the resulting polyesters.^73,74^ In their studies, they found that the molecular weight (MW) of the polyesters was significantly affected by the chain length of the diols, with longer chains generally leading to higher-molecular weights. In these studies, *Candida antarctica* lipase B (CALB), commercially known as Novozym 435, was used as the biocatalyst for the polycondensation reactions. CALB demonstrated broad substrate specificity and excellent stability during the polyester synthesis, leaving no traces of toxic residues. This makes the furan-based polyesters such as PEF biocompatible, making them promising candidates for applications in tissue engineering and other biomedical fields. Comerford et al. also demonstrated the use of CALB as an efficient catalyst for the synthesis of furan-based polyesters. Incorporation of a subsequent thermal treatment step under vacuum conditions significantly enhanced the final molecular weight of the resulting polyesters.^75^ In a recent study, Silvianti et al. reported the one-pot synthesis of poly­(decamethylene-2,5-furanoate) (PDF), a sister polyester of PEF using FDCA and 1,10-decanediol (1,10-DDO) in almost equimolar ratio. The reaction was catalyzed by CALB in the presence of the biobased solvent *p*-cymene, yielding an weight-average molecular weight of 6000 g/mol.^76^ It has been observed that enzyme-catalyzed polymerization of FDCA-based polyesters predominantly employs long-chain diols, such as 1,10-decanediol (1,10-DDO), rather than short-chain polyols like EG, in order to achieve higher-molecular weights. Often enzymes like CALB offer the advantage of stereo selectivity which is an added advantage while copolymerizing with stereo isomers, which is not the case for the MPC process. However, this process also has its drawbacks. For instance, the current process of enzyme-catalyzed polymerization of PEF and related polyesters typically requires high vacuum conditions and low-boiling solvents such as toluene, which are not environmentally benign. Nevertheless, these conditions are often essential to achieve high-molecular-weight polyesters. The efficiency of enzymatic polymerization is sensitive to several factors like temperature, solvent system, water activity, and gradual enzyme deactivation, altogether making the process control challenging at larger scale. Moreover, the high cost of enzymes necessitates efficient multicycle reuse, which presents an additional limitation for process scale-up when compared with the more robust and industrially established MPC process. These challenges of enzymatic polymerization motivate for other emerging “green” alternative, such as ring opening polymerization (ROP), which is an economically competitive technique and possesses promising capability for synthesizing PEF at industrial scale.

#### 2.3.3 Ring-Opening Polymerization

The ROP technique effectively addresses most of the limitations of melt polycondensation that includes high energy consumption, discoloration, byproduct formation, and unwanted degradation. In addition to the principles of “green201d chemistry, ROP also offers the advantage of attaining highly pure PEF suitable specifically for food packaging applications. From a chemistry standpoint, the ROP-based process involves two primary reaction steps: (1) polycondensation followed by depolymerization and (2) ring-opening polymerization. In the first step, DMFDC reacts with EG in an approximately equimolar ratio under highly diluted conditions, using a high-boiling solvent and an inert atmosphere, to form a low-molecular-weight prepolymer known as oligomeric PEF (OPEF). This prepolymer then undergoes depolymerization through intramolecular cyclization, resulting in the formation of a mixture of macrocycles also known as cyclic oligomers (cyOEF).^77^ Figure 5 illustrates the reaction pathway for the synthesis of high-molecular-weight PEF via ROP technique. This cyclization process is favored by the intramolecular cyclization over the intramolecular chain growth at a reaction temperature of around 200 °C and under high dilution condition (10 to 30 g/L) using high boiling solvents like 2-methylnaphthalene.^78^ Among various catalysts employed for this cyclization process, dibutyltin oxide (Bu_2_SnO) is demonstrated to be highly efficient with greater yields of the desired macrocycles. These macrocyclic mixtures of cyclic structure with 2 to 7 rings can be selectively precipitated and separated via filtration under optimized thermal conditions. Detailed investigations into the separation and purification of individual macrocycles have been extensively carried out by Fleckenstein et al.^79^ In the second step, the ring-opening polymerization (ROP) of cyOEF is carried out via a homogeneous melt polymerization process using an appropriate catalyst under an inert atmosphere. Since the melting points of the macrocycles are high (329–350 °C); therefore, a successful ROP of cyOEF requires lowering their melting point below the degradation temperature. This can be achieved by using plasticizers enabling the ROP reaction to proceed at milder temperatures, thus avoiding thermal degradation.

**Figure 5:**
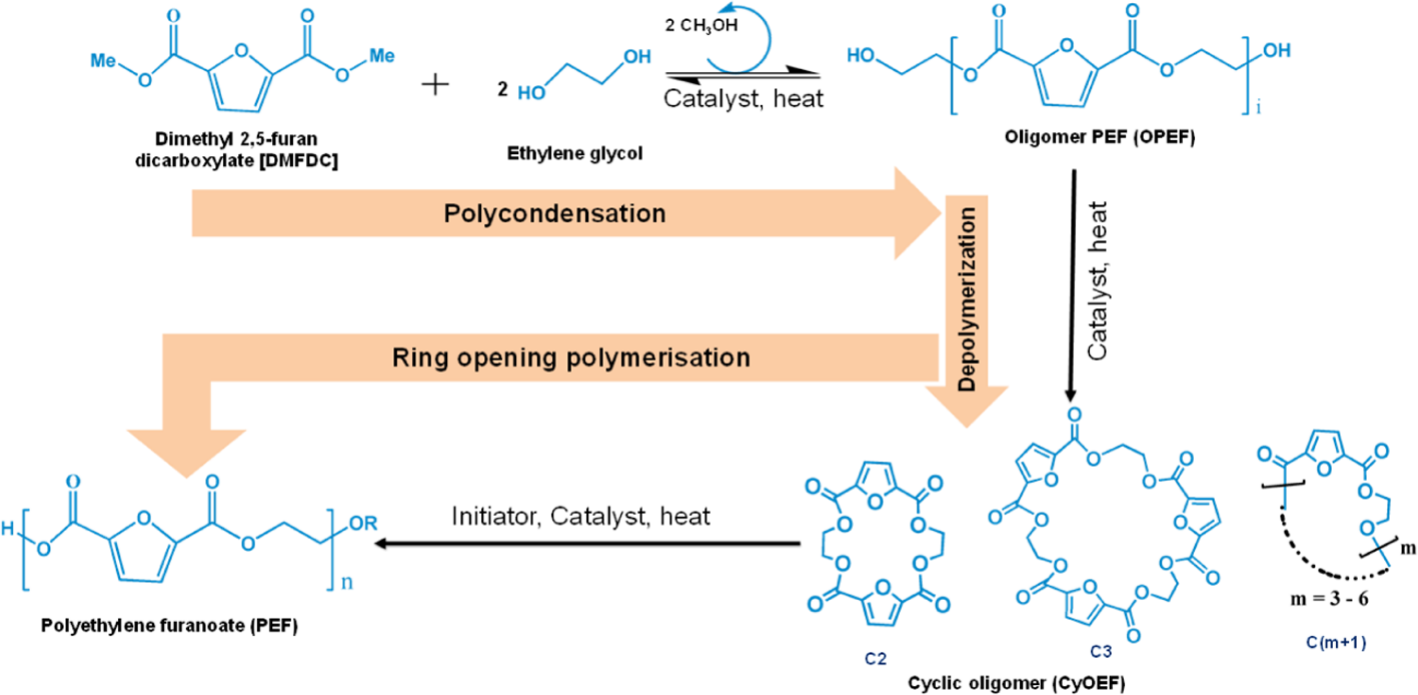
Reaction scheme for synthesis of high-molecular-weight PEF via ROP.

To date, limited studies have reported on the successful ROP of cyOEF at relatively low temperatures under an inert environment using initiators such as cyclic stannoxane or dibutyltin oxide (Bu_2_SnO). The presence of plasticizers like tetraglyme (triethylene glycol dimethyl ether) or tetraethylene glycol dimethyl ether (TEGDME) reduces the viscosity of the reaction melt, thereby enhancing homogeneity as the reaction progresses and ensure effective polymerization. As Hodge and Chakiri explained, the entropically driven ROP of macrocycles such as cyOEF is strongly influenced by the ring–chain equilibrium (RCE) conditions. Establishing well-positioned RCE requires careful optimization of the reaction parameters like the molar ratio of macrocycle to initiator. A higher ratio tends to favor the formation of high-molecular-weight polymers with higher yields.^82^ As discussed above, using the ROP technique for the PEF synthesis presents several challenges. These include difficulties associated with the depolymerization step to obtain cyclic monomers (cyOEF) followed by its tedious purification process. Additionally, the need for high dilution using high-boiling solvents, issues related to plasticization, and the demand for environmentally friendly (green) catalysts further add to the complexity.

Since the ROP technique is highly sensitive to moisture and monomer purity, its industrial-scale implementation that requires highly pure CyOEF is a challenge. Achieving the appropriate cyclic ring size necessary for efficient ROP and the production of high-molecular-weight PEF requires a deeper understanding of the reaction mechanism, kinetics, and thermodynamics, which has not yet been fully established. In addition, monomer chemistry and catalyst selectivity for this reaction have not been explored enough. Successful upscaling needs precise knowledge of process control and reproducibility, areas in which knowledge of ROP-based PEF synthesis is presently very limited. In other words, due to the lack of detailed understanding on complete ROP reaction chemistry and possible complexity in the technology involved at a larger scale, the industrial-scale process is therefore considered riskier than the well-established and mature MPC route. These challenges highlight the need for continued research and development to optimize the ROP process for sustainable PEF production.

## 3 Structure–Property Relationship in PEF

The intrinsic properties of polymers, such as their physical, thermal, mechanical, and chemical characteristics, are fundamentally determined by the chemical structure of the repeating units in their backbone. Additionally, factors such as the spatial conformation of the polymer chains, molecular weight, and crystalline structure significantly influence these characteristics. Such properties form the foundation for evaluating and tailoring polymers for specific end-use applications. Therefore, developing a thorough understanding of the correlation between a polymer’s molecular or physical structure and its resulting properties is essential for designing and optimizing it for the targeted applications. Modification of the molecular structure of polymers through chemical or physical methods can lead to marked changes in the polymer’s performance. Such enhancements are often deliberately performed to tailor polymers for specific applications. Additionally, adjusting processing conditions or applying postprocess treatments can influence both the chemical structure and morphology of the bulk polymer, further influencing its final characteristics. These controlled modifications are frequently employed to expand the range of polymer applications across diverse fields. In other words, the functional property of a polymer is greatly influenced not only by its chemical structure but also by its process conditions and treatments. Figure 6 illustrates the intricate interdependence between polymer structure and properties in special reference to PEF. The structure–property relationships of PEF, along with processing parameters as the third key corner of the relationship triangle, are discussed systematically in the sections below.

**Figure 6:**
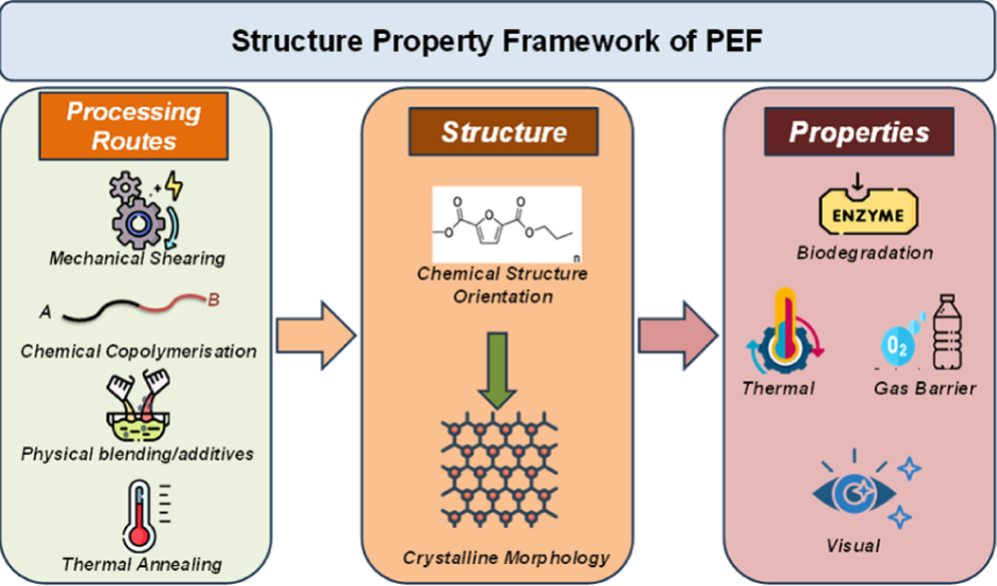
Diagram highlighting the interdependence between the structure and properties of the PEF.

### 3.1 Chemical Structure Influencing Properties

Structurally, PEF can be classified as an “*Alipharomatic polyester*” due to the presence of both aliphatic (ethylene glycol-derived) and aromatic (furan-based) moieties in its backbone. While the resemblance of PEF to PET has been previously discussed, it is the combination of the rigid furan ring and the flexible aliphatic moiety that imparts a unique set of properties in PEF. This structural configuration influences some of the important characteristics such as mechanical strength, thermal stability, and barrier performance. The structure–property relationships of PEF are discussed in detail in this section.

The furan ring in PEF exhibits lower aromaticity than the benzene ring in PET, which is due to the predominant dienic characteristics and presence of an electronegative oxygen atom within the ring. While one lone pair of oxygen participates in π-electron delocalization, the other occupies a p-orbital, thus creating a dipole moment of approximately 0.70 D directed from the ring toward the heteroatom.^83^ Besides, the furan ring being rigid, the head-to-tail interatomic distance between carboxylic acid groups in FDCA (4.83 Å) is shorter than that in TA (5.73 Å).^84^ Unlike the linear geometry of the benzene ring in TA, the furan ring in FDCA is nonlinear and more rigid. Also, a dipole–dipole interaction exists between the polar furan rings of PEF chains. These structural differences contribute to enhanced modulus, tensile strength, and *T*_g_ in PEF compared to PET.^22,85^ This restricted chain mobility also contributes to the inherent brittleness of PEF, reflected in its relatively low average elongation at a break of approximately 3%. While studies have indicated a direct correlation between molecular weight and improved ductility, further systematic investigations are needed to fully elucidate this relationship in PEF.^22^ Besides, the weaker covalent interactions between polymer chains in PEF at temperatures above *T*_g_ contribute to lowering its melting temperature *T*_m_ compared to PET. From the conformational aspects, in PEF, the FDCA moiety can adopt anti or syn, and the EG moiety can take up the *trans* and gauche conformation. In the case of PEF, the interchain interactions promote the formation of an extended zigzag chain conformation during its crystallization.^86^ On the other hand, within the amorphous regions of PEF, the anti^FDCA^–gauche^EG^ conformation has been identified as the most dominant molecular arrangement as evidenced by Fourier Transform Infrared spectroscopy (FTIR) analysis.^87^ Hence, in the amorphous state, PEF chains tend to adopt a randomly coiled-helical conformation. This, in combination with the inherent nonlinear structure of the PEF backbone, aligns with previous observations of a higher free volume in amorphous PEF compared to PET.^88^ The restricted segmental mobility of PEF chains, compensated for by the higher free volume, also explains the higher *T*_g_ of amorphous PEF compared to PET. The gas permeability of a polymer matrix is largely dependent on two basic processes: sorption and diffusion. Lower gas sorption and diffusion are indicative of superior gas barrier properties in polymers. In PET, the benzene ring can rotate freely, which facilitates the penetration and diffusion of gas molecules through the polymer matrix. In contrast, the nonlinear axis of furan ring rotation coupled with its polarity significantly hinders the furan ring flipping.^89^ The restricted furan ring flipping and carbonyl group rotation in PEF, as confirmed by the solid-state nuclear magnetic resonance (NMR) and inelastic neutron scattering (INS) spectroscopy studies, reduces segmental motion of the polymer chains and slows down gas transport.^87,88^ The influence of the higher polarity of the furan ring on the superior gas barrier properties of PEF has been thoroughly investigated by Sun et al.^90^ The rigid furan ring in PEF contributes to a key limitation, i.e., its reduced ductility. The toughness of neat PEF (% elongation at break = 3–5%) falls below the threshold typically required for demanding packaging applications. For example, high toughness and impact resistance are required for beverage bottles, properties that PEF currently underperforms. It is essential to address this drawback, and promising strategies such as chemical modification of the polymer architecture through copolymerization have shown great potential for enhancing ductility and expanding PEF’s application range. The mechanical, thermal, and other relevant properties of PEF are presented in Table 4, with a comparison of the properties of PET.

**Table 4:** A Comparative Data on Various Properties of PEF vs PET

author, year	properties	PET	PEF
Burgess et al., 2014 ^88^	Physical		
	density (kg/m^3^)	1350–1390	1400–1450
Fei et al., 2020 ^91^	Mechanical		
	tensile modulus (Gpa)^a^	2.0–2.5	2.5–2.8
	tensile strength (Mpa)^a^	65–72	67–85
Wang et al., 2017 ^92^	elongation at break (%)	80–200	3^b^
Fei et al., 2020 ^91^	Thermal		
	*T*_g_ ^a^	71–75	82–87
	*T*_m_ ^a^	246–260	210–215
Forestier et al., 2021 ^93^	*T*_cc_	110	160
Ahmed et al., 2025 ^94^	*T*_max_	453	441
Zhao et al., 2021 ^89^	Gas permeability		
	O_2_ (Barrer^c^)	0.093	0.0075
	CO_2_	0.53	-

a The PEF *M*_n_ (10^3^ g/mol) = 83–105, PET = 6.4, PPF = 10.1–30, PBF = 11.8–17.8.

b PEF weight-average molecular weight *M*_w_ = 252,000 g/mol.

c 1 Barrer = 1 × 10^–10^ cm^3^ (STP) cm/(cm^2^ s cmHg); *T*_max_ = Maximum degradation temperature using TGA, 10 °C/min rate, N_2_ atmosphere.

#### 3.1.1 Copolymerization

An excellent review by Terzopoulou et al. in 2020 discusses the advantages of copolymerization of polyesters based on PEF with other polymers that offer advantageous characteristics.^95^ The review refers to copolymerising various aliphatic and alicyclic diacids like sebacic acids,^93^ dodecanoic acid,^96^ and 1,4-cyclohexandicarboxylic acid^97^ improving overall ductility of PEF with a clear sacrifice of tensile strength and modulus. The incorporation of diacids or diols as comonomers can also enhance the ductility of PEF-based copolymers.^98^ Wang et al. used 1,4-cyclohexanedimethylene (CHDM) as a comonomer, and results indicated that the CHDM increased the toughness of the resulting poly­(ethylene-*co*-1,4-cyclohexanedimethylene 2,5-furandicarboxylate)­s (PECFs) without sacrificing the *T*_g_ and barrier properties.^99^ Another group, Xie et al., produced PEF copolymers with tunable ductility ranging from thermoplastics to thermoplastic elastomers (TPE). These tunable physicomechanical properties were composition dependent and synthesized by melt condensation of dimethyl 2,5-furandicarboxylate (DMFDC) and ethylene glycol in the presence of a copolycarbonate diol. With this method, an elongation at break up to 194% was achieved while retaining the tensile modulus within the application range comparable to bottle grade PET (2.2–1.9 GPa). Interestingly, these copolymers also showed a higher CO_2_ barrier performance than PET. By varying the molar percentage of the comonomer, a high-performance thermoplastic elastomer with a high tensile strength and excellent gas barrier was produced. These tunable properties further increase the opportunities of PEF for eco-packaging applications in oxygen-sensitive foods and beverages.^100^ More recently, copolymerization has proven effective in overcoming the inherent brittleness of PEF, enhancing its mechanical flexibility. The flexibility of PEF was improved by incorporating another biodegradable polymer, as shown by Stanley et al.^101^ In their work, they investigated the synthesis and characterization of multiblock copolymers composed of PEF and poly­(ε-caprolactone) (PCL), produced through a two-step melt polycondensation method followed by ring-opening polymerization. PEF–PCL copolymer compositions ranged from 8 to 87% and demonstrated the influence of the plasticizing effect of PCL in reducing PEF’s rigidity. The reduced modulus of three out of the five fabricated copolymers was all below 500 MPa compared to ∼5500 MPa (100% PEF). In addition, a reduction in hardness was also reported for all 5 tested copolymers of <50 MPa, compared to ∼220 MPa for neat PEF. The balance between reduced mechanical strength and improved elasticity can be beneficial for sustainable flexible packaging, influencing both durability and adaptability. The biodegradability of PEF can also be enhanced through copolymerization with biodegradable monomers. Jia et al. synthesized a series of copolyesters, poly­(ethylene dodecanedioate-2,5-furandicarboxylate) (PEDFs), from dodecanedioic acid (DDCA), FDCA, and ethylene glycol. Increasing the content of DDCA unit, the PEDFs were varied from amorphous elastomers to semicrystalline plastics. They found that the FDCA content played a determining role in the biodegradability of the PEDFs over the crystallizability. The PEDFs were all biodegradable, but the degradation rate was determined by their chemical compositions. This study of the enzymatically degradable behaviors of these polyesters is vital to the development of biodegradable products.^96^ In a similar note, extensive studies on the enzymatic degradation of PEF have shown that degradation rates increase with lower crystallinity, elevated temperatures, and the inherently low glass-transition temperature (*T*_g_) of PEF.^102,103^ Random copolymerization of PEF with poly­(ethylene succinate) (PESu) with 50% PEF content forms isodimorphic cocrystallization, which led to a higher degree of enzymatic degradation in comparison to neat PEF.^104^ Details on the enzymatic degradation of PEF are discussed in Section 4.

### 3.2 Morphology Influencing Properties

Semicrystalline polymers such as PEF and PET comprise both crystalline and amorphous phases, whose relative content and morphology are dependent upon various factors. The amorphous phase contributes to optical transparency, while a uniform crystalline phase enhances key properties such as mechanical strength and gas barrier performance, both of which are critical for functional packaging applications. The cooling rate of the polymer melt plays a vital role in determining its final morphology. Generally, rapid quenching favors amorphous structures, while slow and controlled cooling facilitates predominantly semicrystalline morphology. Although PEF exhibits relatively low crystallization propensity, annealing at 160 °C for a longer duration can increase its crystallinity up to 25%.^105^ In general, PEF can crystallize into three polymorphic forms: α (triclinic), ά (pseudomonoclinic), and β (monoclinic), which is a characteristic attributed to the asymmetric and rigid nature of the furan rings in its repeating units.^106,107^ The formation of α and ά polymorphs is highly sensitive to crystallization temperature at around 170 °C. Upon further heat treatment at approximately 190 °C, the polymer chains in the ά form can undergo reorientation to yield more ordered α crystals.^107^ Processing techniques such as extrusion, injection molding, stretch blow molding, and thermoforming inherently involve stretching and shearing, which influence crystallization kinetics and morphology of polymers.^109^ Manipulating the crystalline morphology of PEF through adopting various processing techniques, uniaxial stretching, the addition of nucleating agents, or the incorporation of platelet-like nanomaterials has been shown to significantly improve functional properties of PEF. Such changes in the crystal structure also led to improvement in thermal properties such as *T*_m_, *T*_g_, and *T*_c_ of PEF. PEF also exhibits strain-induced crystallization (SIC), whereby the amorphous phase transforms into a mesophase under critical strain at elevated temperatures of 200 °C.^110^ These mesophases act as physical cross-link sites within the polymer matrix, which enhances the mechanical performance of PEF under load-bearing conditions, significantly enhancing mechanical stability under load-bearing conditions.^111,112^ Details about the crystalline nature of PEF under various conditions are studied elaborately.^105^ In the crystalline regions, the unit cell packing density of PEF (1.56 g/cm^3^) is higher than equivalent PET (1.45 g/cm^3^), which is why PEF shows higher gas barrier property.^87^ Although the term biodegradability is a promising feature for polymers, especially with the drive for sustainability, it can be misleading as the degradation rate can be slowed due to unsuitable conditions.^113^ The crystalline phase in PEF hinders the hydrolysis of polymer chains and thereby reduces the rate of biodegradation. The literature is scant to understand the real behavior of PEF under composting, soil or marine aquatic environments, details of which is discussed in the Section 4.4 organic recycling.^26^ Nonetheless, PEF remains a promising candidate for enzymatic degradation, as demonstrated by Dolz et al., who recently developed a high-throughput colorimetric screening assay to help identify PEFases for future work in this area.^114^

Physical modification: it is an easy and effective approach to enhance the performance of PEF by altering its morphology, crystallization behavior, interfacial interactions, and free volume distribution, without modifying its fundamental chemical structure. Designing optimized blends of PEF with other polymers or developing composites and nanocomposites using various micro- or nanofiller offers a practical route to tailor and enhance its properties for specific applications. Poor UV stability is a known issue with PEF when compared to PET. Maaskant and van Es reported that PEF shows severe signs of degradation when exposed to UV irradiation, including discolouration, chain scission, cross-linking, and a reduction in its *T*_g_ as well as its ability to crystallize.^115^ Blending PEF with PET can enhance the UV stability of PEF, significantly reducing these issues. Through differential scanning calorimetry (DSC) analysis, Poulopoulou et al. demonstrated that PEF is immiscible with polycarbonate (PC) and PLA, while showing good miscibility with structurally related furanoate-based polyesters such as poly­(propylene furanoate) (PPF) and poly­(butylene furanoate) (PBF).^116^ In another study, Fredi et al. showed that blending PEF (10 wt %) in the PLA matrix in the presence of commercial compatibilizer Joncryl ADR 4468 increases the compatibility between the two polymer phases, which was evidenced by reduced droplet size, increased complex viscosity, and improved mechanical properties. The presence of PEF in this optimized blend offered significant improvement in O_2_ gas barrier and UV stability compared to neat PLA.^117^ Electrospun nanofibrous membranes (ENMs) with a 40:60 PEF to PLA ratio were identified as the optimal composition, exhibiting exceptional water flux across various pressures, making them well-suited for water filtration applications.^118^ In PEF/poly­(butylene adipate terephthalate) (PBAT), blends with 1–50 wt % of PEF content has been shown to develop immiscible, but compatible blend systems. The addition of PEF does not change the crystallization behavior of PBAT; however, the resulting sea–island morphology of PEF domains dispersed within the PBAT matrix increased the blend’s toughness. These findings highlight the potential of PEF to synergise with other biobased polymers for various applications.^119^ As previously mentioned, in the melt polycondensation route for PEF synthesis, an SSP step is essential to achieve high-molecular-weight blow-molding-grade PEF. A fast crystallization rate is advantageous during SSP as it facilitates an increase in molecular weight. Moreover, for nontransparent applications, faster crystallization can contribute to improved heat resistance. To increase PEF’s rate of crystallization, cellulose has been incorporated as a reinforcing phase in thin-film PEF/cellulose composites. Cellulose acts as a nucleating agent within the PEF matrix and enhances the crystallization rate as its content increases.^120^ A recent study by Han and Liao demonstrated that a PEF/poly­(glycolic acid) PGA/PCL blend, with a fixed PGA/PCL ratio of 75/25, forms a compatible blend system with higher optical transparency and significantly improved biodegradation performance compared to neat PEF.^121^

### 3.3 Processing Condition Influencing Properties

The correlation of processing conditions on the polymer morphology is illustrated in Figure 6 and emphasizes the importance of controlling morphology to tailor material properties. For instance, there is research that shows the effect of annealing on the crystallinity of PEF.^108,122,123^ Pluta et al. found that when annealing PEF, the crystallization temperature (*T*_c_) influenced the degree of crystallinity. At a *T*_c_ of 120 °C, crystallinity was measured to be 28.7%; increasing *T*_c_ to 180 °C increased the degree of crystallinity to 41%.^122^ Codou et al. found that PEF displays faster crystallization rates during glass crystallization compared to during melt crystallization. They found slower kinetics of melt crystallization, which may be due to random secondary nucleation that inhibits the lateral growth of the crystalline phase. The faster kinetics of glass crystallizations may be due to the more dominant role of primary nucleation at lower temperatures.^123^ The kinetics of PEF during melt and glass crystallization are important for industrial applications where slow crystallization is more desirable in blow and injection molding. Research on the influence of various processing conditions on the final properties of PEF is not sufficient to draw definitive conclusions. This void in understanding is primarily due to the fact that PEF is a relatively new polymer and industrial scale production of PEF is very limited to understand impact of processing behavior at large-scale processing setup. Further research is necessary to fully understand the behavior of PEF under different processing conditions, which is critical for optimizing performance.

## 4 Recycling of PEF

The plastics waste management hierarchy is a cornerstone of sustainable practices, which emphasizes minimizing plastics led environmental pollution. Unlike the conventional pyramid hierarchy, this framework is designed as an inverted pyramid consisting of six components, starting from the top: refuse, reduce, reuse, recycle, recover, and finally disposal, as shown in Figure 7. Refuse: avoid waste creation by turning down unnecessary or least important plastic products; reduce: minimizing the use of plastic products; reuse: repeated use to enhance the service life; recycle: processing waste plastics into new products; recover: energy recovery or composting of plastics products; disposal: landfilling and incineration are less favorable option of plastics waste management. This hierarchy prioritizes upstreaming of plastics products over downstream solutions, to promote circular economy.

**Figure 7:**
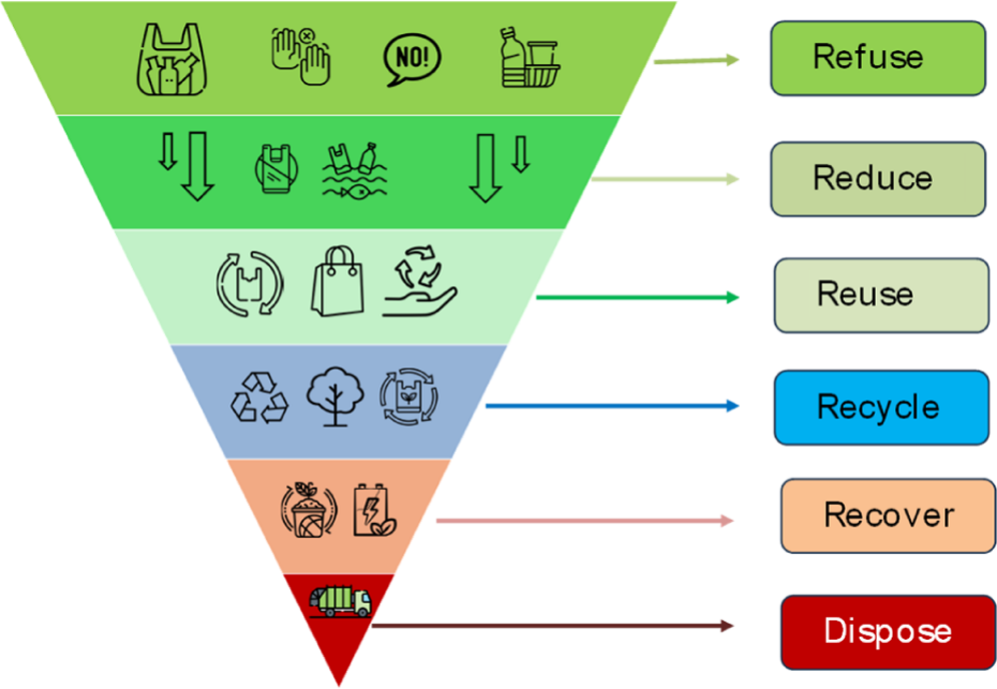
Inverted pyramid framework illustrating the waste management hierarchy in descending order of priority: refuse, reduce, use, recycle, recover, and disposal.

As PEF production is expanding, understanding its end-of-life management has become more crucial. Aligning the end-of-life strategies of the PEF with the principles of the inverted pyramid framework will be essential to ensure its sustainable integration into the market. As PEF has received diverse application possibilities across multiple sectors, its recycling is likely to become complex due to variations in composition, contamination, and limitations associated with efficacy in material separation. Studying these processes early in PEF commercialization is essential ensuring sustainable EoL practices. To that end, it is vital to explore the opportunity for mechanical, chemical, and biological recycling of PEF in different forms.

### 4.1 Mechanical Recycling

The mechanical recycling refers to the melting and reprocessing of polymers into new products. It is the cheapest and simplest way to recycle plastics. The mechanical recycling of plastics typically leads to a downcycling into lower-grade materials or blending with virgin material to generate products of similar quality to the original materials. The chemical structure of PEF resembles that of PET, which makes them comparable in various aspects, including recycling processes. Being fully biobased, the mechanical recycling of PEF is interesting in the broader context of sustainable waste management. PEF can be processed in the same facilities as PET, which provides the key advantage of utilizing existing infrastructure. Furthermore, studies indicate that cross-contamination of PET with up to 2 wt % PEF does not compromise the quality of recycled rPET even after reprocessing.^84^ However, mechanical recycling or repeated melt reprocessing of PEF leads to polymer chain scission through hydrolysis under the influence of a high temperature and mechanical shear. The degradation not only impacts reprocessing behavior but is also highly detrimental to overall performance of PEF. Several studies indicate that optimization of process parameters, blending with other polymers, and incorporating additives can significantly enhance the mechanical recycling process of PEF. However, if PEF ends up in landfills, it is likely that these additives could leach out into the environment, and their impact should be assessed.^124^ Table 5 presents a summary of various studies on the mechanical recycling of PEF and its blends which outlines the type of study conducted and the derived key findings.

**Table 5:** Summary of Various Studies on the Mechanical Recycling of PEF and Its Blends

material	type of study	knowledge outcome	ref
PEF	study on effect of titanium or tin-based catalysts on synthesized PEF and understanding its mechanical recycling feasibility	tin-based catalysts are advantageous for mechanical recycling of the synthesized PEF, keeping thermal properties constant even after three consecutive recycling	^63^
PEF	Paszkiewicz et al. reported the influence of the three consecutive injection molding followed by palletization on intrinsic viscosity, mechanical property of PEF.	the second and third cycle of recycling caused chain scission resulting in reduced intrinsic viscosity and deteriorates the mechanical properties significantly	^125^
PEF blends	investigation of the morphology, thermal properties, crystal structures, and mechanical properties of novel biobased high toughness PBAT/PEF blends.	PBAT and PEF were compatible, and PBAT/PEF blends were successfully prepared by a melt blending method. The PBAT/PEF blends were pure physical blends, displaying a typical sea-island morphology and maintaining toughness and high elongation even after recycling. The blends had good thermal stabilities and mechanical properties	^119^
PEF blends	synthesis of furandicarboxylic Acid (FDCA) to produce PEF, a biobased alternative to PET.	results revealed that up to 5% (w/w) PEF has no significant effect on the mechanical and physical properties of PET. Tensile 148test showed that there was no negative influence of small additions of PEF to a PET stream. PEF can compete with PET on both price and performance, and with a significantly better environmental footprint	^32^
PEF blends	the mechanical recycling of partially biobased and recycled PET blends by reactive extrusion with poly(styrene-*co*-glycidyl methacrylate)	the original biopolymer properties can be successfully restored, and the ultimate performance of bio-PET articles would be retained for a given number of reprocessing cycles. Mechanical recycling for biobased but nonbiodegradable polymers will be appropriate from both an economic and environmental point of view, thereby accelerating the transition of the plastic packaging industry from its traditional linear model to a more valuable and sustainable circular model	^126^
PEF blends	investigation of the structure, thermal properties, and the miscibility of a series of poly(ethylene terephthalate)/poly(ethylene 2,5-furandicarboxylate) (PET/PEF) blends	the presence of eco-friendly PEF, originating from renewable resources in the blends, enables a reduction of the environmental impact from the production of fully-PET based products. The recyclability of the ultimate products remains high. Blends are expected to play a major role for the increase in sustainability and the transition to ‘green’ economies	^127^

Figure 8 presents the comparison of mechanical, chemical, and enzymatic recycling approaches for PEF based on technical maturity and economic feasibility. Mechanical recycling is the most technically advanced and cost-effective method currently available. Chemical recycling is classified further into acidic, neutral, and alkaline processes, which are more mature and scalable than enzymatic routes. Enzymatic recycling is still in its early stage of development and requires optimization for industrial application. Chemical recycling is a recognized process for addressing the limitations of mechanical recycling particularly for complex and contaminated plastic waste. Unlike mechanical recycling, it breaks down polymers into their original monomers and allows resynthesis of high-purity materials. For PEF, chemical recycling is especially promising as it enables preservation of PEF’s superior barrier, mechanical, and thermal properties across multiple lifecycles, making it a key component of future sustainable EoL strategies.

**Figure 8:**
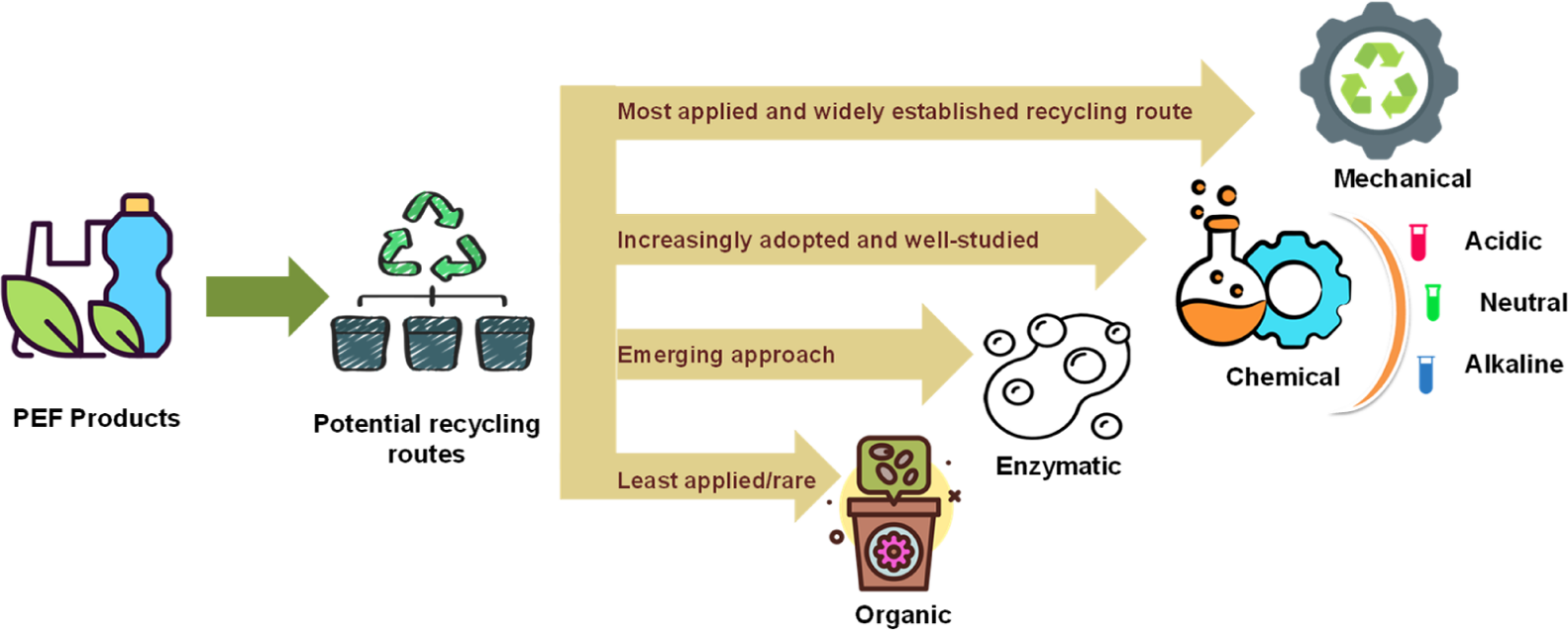
Comparison of PEF recycling methods based on technical maturity and economic feasibility.

### 4.2 Chemical Recycling

Chemical recycling of PEF shares similarities with PET, and both polymers can be depolymerized into their respective monomers under specific conditions. The primary degradation pathway for PEF is hydrolysis, which leads to the recovery of FDCA which is the primary monomer and EG. Beyond hydrolysis, advanced recycling techniques, such as glycolysis, methanolysis, and enzymatic degradation, offer additional routes for breaking down PEF into valuable intermediates. Understanding these processes is crucial for developing efficient recycling strategies that minimize environmental impact while maximizing material recovery. This section will explore the various chemical recycling technologies applicable to PEF, highlighting their mechanisms, efficiencies, and potential for industrial adoption. However, reports on these methods are limited due to the relatively recent emergence of PEF as a commercial material and the lack of extensive studies on its long-term degradation behavior and scalability.

#### 4.2.1 Hydrolysis of PEF

Hydrolysis is one of the most extensively studied methods for the chemical recycling of polymers. It involves the breakdown of polymer chains through their reaction with water, typically under neutral, alkaline, or acidic conditions, depending on the pH and the nature of the catalyst used. While hydrolysis is an effective approach for polymer depolymerization, it also presents several limitations, including the requirement for high reaction temperatures and, in some cases, extremely high pressures to achieve complete depolymerization. Similar to PET, the hydrolysis of PEF can be classified into three categories: neutral, alkaline, and acidic. Each of these will be discussed in the following sections.

#### 4.2.2 Neutral Hydrolysis

Neutral hydrolysis of PEF involves breaking down the polymer into its monomeric components using water under neutral pH conditions. This process yields FDCA and ethylene glycol, both of which can be repurposed for new PEF syntheses, aligning with circular economy principles. Hydrolysis is carried out using only water, under high temperature and pressure. This method avoids the use of strong acids or bases, making it a more environmentally friendly approach.^128^ Temperatures above 200 °C significantly enhance the hydrolysis rate, leading to an efficient polymer breakdown. Although direct studies on the neutral hydrolysis of PEF are scarce, existing research on PET offers a valuable foundation for exploring potential pathways for PEF depolymerization under similar conditions. For instance, a study by Martin et al. demonstrated that neutral hydrolysis of PET can be effectively catalyzed by ionic liquids.^129^ Specifically, terephthalate-based ionic liquids facilitated the hydrolysis of PET at 200 °C, achieving a 94% yield within 4 h. Given the structural similarities between PET and PEF, it is plausible that similar catalytic approaches could be adapted for PEF depolymerization under neutral conditions. Therefore, further research into the application of ionic liquids for PEF hydrolysis could provide deeper insights into the mechanisms and efficiency of PEF’s neutral hydrolysis.

#### 4.2.3 Alkaline Hydrolysis

Alkaline hydrolysis is a well-established depolymerization method of PET, utilizing strong bases such as sodium hydroxide (NaOH) or potassium hydroxide (KOH) to cleave the ester bonds of polymer chain.^130^ Similarly, alkaline hydrolysis of PEF has demonstrated promising results under optimized conditions, effectively breaking down the polymer into its constituent monomers, FDCA and EG. A recent study by Wang et al. demonstrated the feasibility of chemically depolymerising PEF through alkaline hydrolysis with efficient and high-quality recovery of its monomer. Although alkaline hydrolysis typically takes place under a moderate to high-temperature range (60–160 °C), optimal reaction conditions for nearly complete PEF conversion (99.0%), high FDCA yield (85.6%), and high FDCA chemical purity (98.7%) were achieved at an elevated temperature of 160 °C. A relatively high NaOH-to-PEF molar ratio of approximately 6.6:1 and optimization of the reaction time between 30 min to 2 h were necessary for attaining the maximum yield of FDCA.^131^ Under similar reaction conditions, alkaline hydrolysis of PEF is significantly faster than PET, indicating that the structure of PEF allows for easier breakdown under alkaline conditions, which is stated to be due to enhanced susceptibility of the furan ring toward hydrolysis compared to the aromatic ring in PET. As in the case of PET, the crystalline regions in PEF hinder depolymerization, requiring longer reaction times or more aggressive conditions to achieve complete breakdown, though the effect is less pronounced due to its inherent reactivity of furan rings.^132^ Recently, Dargó et al. demonstrated that PEF being partially soluble in MeSasamol at room temperature undergoes enhanced depolymerization under basic conditions containing potassium hydroxide (KOH) and methanol. The presence of MeSasamol as a cosolvent increased the PEF conversion at room temperature up to 95%, making it environmentally friendly and economically viable process for PEF depolymerization.^133^

#### 4.2.4 Acidic Hydrolysis

Acidic hydrolysis is a common method for breaking down polyesters like PET and involves using strong acids, such as sulfuric acid or hydrochloric acid, to cleave the ester bonds within the polymer backbone. This process typically occurs under high temperatures and pressures, leading to the formation of monomers or oligomers that can be further purified and reused. The reaction rate is influenced by factors, such as acid concentration, reaction time, and polymer crystallinity. However, despite its success in PET bottles and other polyester recycling, there is limited research available on the application of acidic hydrolysis to PEF.^134^ The use of strong mineral acids in the acidic hydrolysis process does not align with the sustainability aspects associated with PEF, rendering this approach as less suitable for further research. Most studies on PEF depolymerization have focused on enzymatic and alkaline hydrolysis, demonstrating significant efficiency in breaking down the polymer under controlled and environmentally sustainable reaction conditions. The lack of studies on acid-catalyzed hydrolysis suggests a gap in current research, and further investigation is needed to determine its feasibility, efficiency, and potential advantages over other methods.

#### 4.2.5 Glycolysis of PEF

Glycolysis is another well-established degradation process commonly used for depolymerization of polyesters like PET. Glycolysis of PEF leads to formation of EG and bis­(2-hydroxyethyl)-furan-2,5-dicarboxylate (BHEF) which are the monomers. While hydrolysis typically yields only monomers, glycolysis can result in a mixture of monomers and oligomers.^135^ This process is widely utilized for recycling clear PET bottles, and studies have demonstrated its feasibility for PEF as well. Among all other techniques, glycolysis is the most favored chemical recycling process for PEF due to its mild reaction conditions and the ease of separating the monomers.^136^ Hence, the production of monomers through glycolysis presents a promising pathway for closing the loop in PEF recycling, contributing to a more sustainable circular economy. Gabriondo et al. reported an effective method of depolymerization of compression-molded PEF films by glycolysis using an organo-catalyst. Their results showed that benzoic acid (BA) was not able to depolymerize PEF into BHEF, but basic catalysts of 1,8-diazabicyclo­[5,4,0]­undec-7-ene (DBU) and 1,5,7-triazabicyclo­[4,4,0]­dec-5-ene (TBD) gave yields of 55% and 72%, but the mixed catalyst of DBU/BA resulted in a yield of 92%. DBU/BA is water-soluble and could easily be removed and recovered to be reused in the study. While the depolymerization of PEF has been demonstrated successfully in this study, it still requires relatively high temperatures for efficient breakdown. Increased reaction time, temperature, and catalyst loading up to 5 wt % offer advantage of higher percentage yield of monomers. The calculated activation energy of 163 kJ mol^–1^ from the study further highlights the high energy input necessary for depolymerization with higher yield, which could present a challenge for industrial-scale recycling.^137^ Significant research efforts are currently focused on understanding the glycolysis of PEF to achieve high-purity and high-conversion rates for the production of its monomer by varying parameters such as catalysts and employing microwave-assisted heating.^136,138^

#### 4.2.6 Alcoholysis of PEF

Alcoholysis involves the depolymerization of PEF in the presence of alcohol as the solvent under high temperature and high pressure. The alcoholysis of polyester consists of breaking the backbone ester linkage through an attack of alcohol (e.g., methanol or ethanol) on the carbonyl group, known as a transesterification reaction, leading to the formation of monomers. Within alcoholysis, methanolysis seems to have gained the most amount of attention over recent years and is often used in recycling PEF. The use of other alcohols has been reported in PET depolymerization such as ethanol and butanol. In a study conducted by Alberti et al., the methanolysis of PEF was investigated using 1,5,7-triazabicyclo­[4,4,0] dec-5-ene (TBD) as an organo-catalyst at a concentration of 10.7 mol %. The depolymerization was carried out at 90 °C for 30 min with 63% yield of DMFDC. Note to mention that DMFDC and EG are the key monomer pairs in the industrial synthesis of PEF. Later, the same research group reported a method for a microwave-assisted methanolysis for the depolymerization of PEF using Zn­(OAc)_2_ as a catalyst under microwave heating at 120 °C with yield of 38%.^139^ The reaction was then scaled up, and the study reported achieving 90–92% NMR yields for 1 and 5 g of PEF reactions, with successful crystallization and purification of the depolymerized product. This method also demonstrated feasibility in mixed-polymer systems, allowing selective depolymerization without preseparation. Furthermore, the depolymerization products from this report were successfully repolymerized into PEF, achieving 81% yield. This approach presents an efficient route for recycling PEF, offering a sustainable end-of-life solution through catalyst-assisted depolymerization and polymer regeneration. The use of microwave-assisted depolymerization in this report enables depolymerization to occur in minutes rather than hours, making it ideal for scalable, high-throughput processes. Although the methanolysis process is not fully established for PEF, the process is well-established for PET, where it effectively depolymerizes PET into dimethyl terephthalate (DMT) and EG. For instance, a study demonstrated a low-energy catalytic route for the methanolysis of PET, achieving a 93.1% yield of DMT at ambient temperatures.^140^ Given the structural similarities between PET and PEF, it is plausible that methanolysis could be applied to PEF as well. However, specific studies focusing on the methanolysis of PEF are currently scarce.

### 4.3 Enzymatic Recycling

Enzymatic depolymerization of polymers is one of the most sustainable and environmentally friendly approaches to chemical recycling. Unlike traditional chemical methods, enzymatic recycling operates under milder conditions, reducing energy consumption and minimizing the use of harsh chemicals. This process relies on specialized enzymes, primarily esterases and cutinases, which catalyze the hydrolytic cleavage of ester bonds in PEF, breaking it down into its monomer components of FDCA and EG. The enzymatic recycling technology is evolving rapidly and is increasingly recognized either as a specialized branch of chemical recycling or as a distinct recycling category, separate from conventional chemical and mechanical processes. Pellis et al. investigated the enzymatic depolymerization of PEF with varying molecular weight using *Thermobifida cellulosilytica* cutinase 1 (Thc_Cut1), which is an enzyme that degrades PET effectively. Thc_Cut1 exhibited efficient catalytic activity across all six PEF variants, with hydrolysis conducted over 72 h at 50 °C. Notably, the highest hydrolytic activity was observed in case of PEF with molecular weight of 40 kDa, yielding 13 mmol L^–1^ FDCA monomer.^141^ Typically for PEF hydrolysis, lower-molecular-weight polymers tend to hydrolyze faster, possibly due to difference in the PEF crystallinity. Subsequently, in a comparative investigation on enzymatic hydrolysis of PEF and PET performed by Weinberger et al., it was observed that hydrolysis of PEF is significantly faster than PET. In this study, under identical reaction conditions, two different enzymes namely, *T. cellulosilytica* cutinase 1 (Thc_Cut1) and *Humicola insolens* cutinase (HiC) were utilized as biocatalysts. The PEF exhibited a higher susceptibility to enzymatic degradation than PET, as evidenced by measurement of mass loss using quartz crystal microbalance (QCM), increased surface erosion and roughness through scanning electron microscopy (SEM) and atomic force microscopy (AFM) analysis of surface, and significantly greater release of FDCA relative to TA.^103^ The findings of this research highlight PEF’s superior enzymatic degradability compared to PET, reinforcing its potential as a more sustainable alternative in polyester applications. Another study carried out by Dolz et al. investigated cutinases and related enzymes from organisms like HiC and Thc_cut1 as they have shown promise in breaking down PEF.^114^ The primary objective of this study was to validate a colorimetric high-throughput screening application (HTS) assay for enzymatic PEF degradation based on PEF soluble scaffolds. The goal was to optimize these enzymes through directed evolution to improve their ability to degrade PEF. Recently, Kumar et al. used two highly efficient PET hydrolases, namely, Fast Polyester Hydrolase (FastPETase) and leaf branch compost-cutinase to understand the depolymerization behavior of PEF under similar reaction conditions. FastPETase is an engineered enzyme designed to efficiently degrade PET under mild conditions. In the study, leaf branch compost-cutinase exhibited superior performance, achieving 98% depolymerization within 72 h at 65 °C, while FastPETase reached a 78% weight loss under controlled conditions, improving to 92% when the substrate concentration was reduced. The primary hydrolysis products included FDCA, ethylene glycol, and mono­(2-hydroxyethyl)-furanoate (MHEF). However, it can be observed that the presence of crystalline PEF significantly hindered degradation. Microscopy studies carried out by the group confirmed extensive surface erosion of amorphous regions while highlighting the resistance of crystalline phases to enzymatic attack. These findings give crucial insights into the enzymatic breakdown of PEF and give insight into efficient biocatalytic recycling strategies.^132^ The feasibility of enzymatic depolymerization of PEF is highly influenced by factors such as polymer crystallinity, enzyme specificity, and reaction conditions. Studies have shown that cutinases, particularly those engineered for enhanced activity, can effectively hydrolyze PEF but at slower rates compared to PET due to the structural differences between the two polymers. However, PEF’s biobased origin and its enhanced hydrolytic degradability compared to PET make enzymatic recycling a viable end-of-life strategy, especially when coupled with optimized process conditions and enzyme engineering advancements.

While PET benefits from well-established recycling infrastructures, similar efforts must be needed toward PEF to fully assess the environmental benefits of its EoL management through recycling.^142^ The ability of PEF to be recycled on a large scale is heavily dependent on the development of collection and recycling systems. Due to visual similarities between PEF and PET, additional measures such as clear and distinctive labeling will be needed to ensure effective separation. The behavior of PEF in chemical recycling at a large scale is still uncertain. Advancing efficient chemical recycling methods for PEF is essential to fully realize its environmental benefits, decrease dependence on fossil-derived plastics, and support the transition toward a truly circular economy.

### 4.4 Organic Recycling

Organic or biological recycling refers to the use of microorganisms to break down materials into valuable resources, such as compost or biogas, under controlled conditions. During this process, materials initially break down to smaller compounds, which then mineralize into their elemental components like carbon, nitrogen, oxygen, and sulfur via. natural biogeochemical cycles. It is the final and crucial step in a sustainable waste management hierarchy that ensures the safe disposal of materials. There are two methods that involve organic recycling: aerobic digestion (in the presence of oxygen) and anaerobic digestion (in the absence of oxygen), both of which ultimately convert plastics into gases and moisture as end products. Early studies until 2015 did not establish whether PEF was biodegradable plastic. In 2020, Avantium, in collaboration with Organic Waste Systems (OWS), Belgium suggested that under controlled composting conditions, PEF satisfies the term “Biodegradable” as per standard ISO17088:2012.^143^ In a field trial experiment performed by Avantium, Amsterdam reported 90% biodegradation of amorphous and oriented PEF within one year.^26^ As with other biodegradable polyesters such as PLA, the degradation of PEF is believed to be initiated by hydrolytic cleavage of its ester bonds at the polymer–water interface. However, the revised standard ISO 17088:2021 introduces updated test procedures and requirements for plastic biodegradation, necessitating a reassessment of the previous report by the OWS. Since then, no systematic studies have confirmed PEF’s biodegradability in realistic environmental conditions. It was pointed out in a recent review by Silverwood et al. that further work is needed to understand how home or industrial composting can be utilized as an end-of-life pathway for PEF.^113^ In other words, there is no clear report that says PEF is biodegradable or compostable like PLA, PCL, or PHAs. Hence, authors strongly believe it is too early to categorize PEF in “biodegradable” or “nonbiodegradable” materials and would recommend the necessity for more research on this aspect.

## 5 Applications and Sustainability

### 5.1 Applications of PEF

The fundamental reason for the growing interest in PEF’s multifaceted applications over PET is its 100% biobased nature, technically aligning itself with the UN’s sustainability development goals. Other than PEF being renewably sourced, the energy consumption and GHG emission during production of PEF from well-established corn-based fructose is found to be significantly lower compared to the production of PET.^27^ Also, the GHG emission for PEF production is relatively much lower compared to the production of PLA, PHA, and Bio-PE from renewable sources.^27^ A study reported by Jiang et al. demonstrated production of PEF from industrially captured 99.5% pure CO_2_ and biowaste material as primary feedstock, which not only reduced GHG emission but also reduced the energy usage by 40.5% compared to the PET production process.^67^ Consumers are the key to the implementation of sustainability across the globe, with 78% of global consumers aspiring to environmental sustainability. Moreover, 63% of global consumers have made modest to significant shift in their consumption behavior toward being sustainable.^144^ This favorable sentiment supports the introduction of PEF as a biobased, sustainable alternative to PET. One of the most significant properties of PEF is its superior gas barrier performance compared to PET, extending product shelf life offering important advantages for the food and beverage and cosmetic and medicine industries.^26,137,145^ PEF also shows a higher tensile strength, tensile modulus, and storage modulus which allows less material usage in comparison to PET for packaging applications that require enhanced structural integrity.^88^ The nonlinear furan ring and its dipolar characteristics along with the hindered ring-flipping mechanism are some of the fundamental factors influencing the mechanical properties of PEF, and the mechanism is discussed in Section 3 of this review. Considering its favorable gas barrier, thermal and mechanical properties along with its processability advantages, PEF demonstrates significant potential as a sustainable alternative to PET for applications particularly within the domain of packaging and textile sector. The infographic in Figure 9 illustrates the broad range of current and emerging application areas of PEF in various forms, highlighting its potential to replace PET and contribute to sustainable innovation. The applications include advanced packaging, textiles, biomedical, electronics, and optoelectronics and 3D printing each of which is discussed in detail herein.

**Figure 9:**
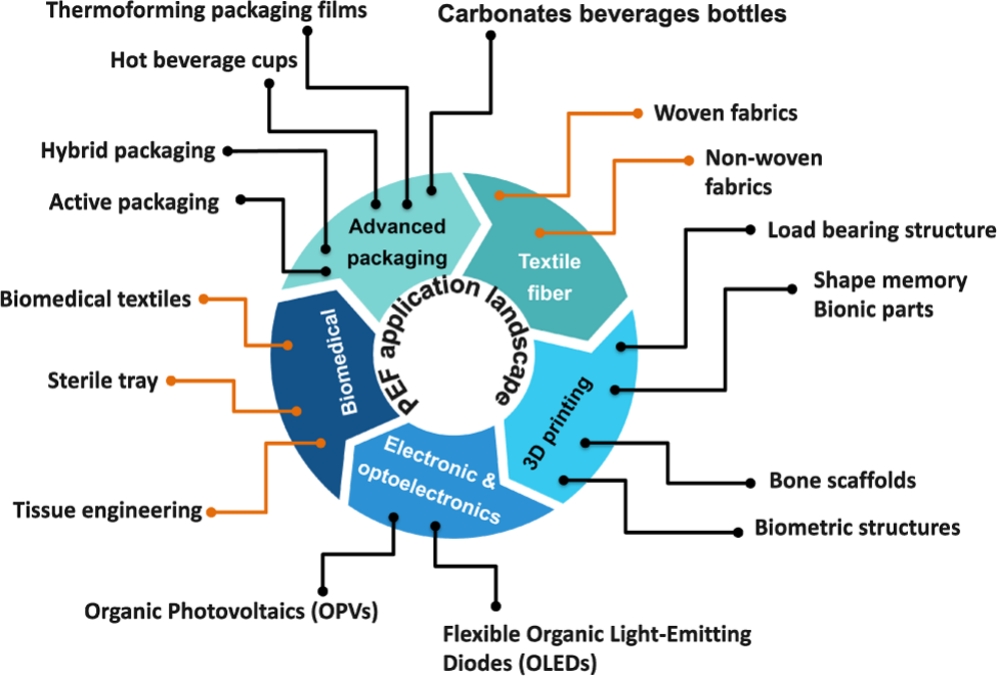
Application landscape of PEF.

### 5.2 Advanced Packaging Application

PET was introduced as an economical alternative to glass by DuPont in 1973. Since then, the global beverage industry has grown to over 500 billion PET bottles annually.^146^ The beverage industry uses coatings, additives, or nylon layers to improve the barrier property of PET which complicates the PET recycling process and increases cost.^147^ On an industrial scale, PEF-based bottles can be more energy efficient due to its lower *T*_m_, high melt strength, while also needed less material mass per product compared to PET bottles.^26,91^ Biaxial orientation is a widely used processing technique to improve polymer properties for packaging applications such as the stretch blow molding of beverage bottles. A comparative study by van Berkel et al. showed that biaxially oriented PEF, when processed with an appropriate stretch ratio, exhibits a higher modulus and more consistent mechanical performance than PET.^148^ Similar to PET, uniaxial stretching of PEF above its *T*_g_ enhances its overall stretchability, which is attributed to the reorganization of its crystalline microstructure.^110^

### 5.3 Active Food Packaging

The food industry is continuously struggling with the demand from consumers for longer food shelf life, which not only contributes to world food security by reducing food waste but also benefits the environmental aspects through lowering CO_2_ emission. Active and intelligent packaging assures high quality and extended shelf life of food products, which has gained enormous attention in the post COVID-19 pandemic era. Active or intelligent packaging is a broad term that includes properties like antimicrobial properties, antioxidant properties, freshness indicators, high moisture barriers, etc. These features are achieved by incorporating active agents such as nanoparticles or biomolecules, which releases the active agents as the functional element. To improve antibacterial properties of PEF, Zhu et al. made PEF/ZnO thin films using a solvent casting method.^149^ PEF/ZnO and PEF/modified (m)-ZnO films demonstrated strong antimicrobial activity against *Escherichia coli*, achieving bacteriostatic rates of 93.4% and 97%, respectively. The modified surface of the NPs was fabricated by reacting ZnO with (3-aminopropyl)­triethoxysilane (APTES), increasing their hydrophilicity. The enhanced performance of *m*-ZnO films was attributed to improved nanoparticle dispersion and surface modification. This antibacterial effect is primarily driven by reactive oxygen species (ROS) generation rather than Zn^2+^ ion release or mechanical disruption. Notably, high antibacterial efficacy was maintained even at low ZnO concentrations (below 10 wt %) across a range of polymer/ZnO coatings. More recently, Stanley et al. also developed three different PEF nanocomposites incorporating Ce-bioglass, ZnO, or ZrO_2_ nanoparticles that were synthesized via in situ polymerization.^150^ Compared to neat PEF, all nanocomposite samples demonstrated a higher percentage of inhibition against *Staphylococcus aureus* and *E. coli*. The antimicrobial efficacy of the three nanocomposites ranged from 9–22% when tested against *S. aureus* and 5–16% when tested against *E. coli*. PEF-Bioglass was more effective against Gram-positive *S. aureus*, while the zinc-based additives worked better against *E. coli*. ZnO composites also exhibited enhanced hardness and elastic modulus, while all formulations retained low-to-moderate color shifts. In similar work by Stanley et al., silver nanoparticles were added to make a PEF-based nanocomposite that showed up to 30% death of *E. coli*.^150,151^ However, the authors did state that with further optimization, the fabricated nanocomposites could be used in secondary packaging. These findings underscore the potential of tailored PEF-based nanocomposites as effective antimicrobial packaging solutions, offering enhanced mechanical performance with a minimal impact on appearance.

### 5.4 Hybrid Packaging

Hybrid or multilayer packaging plays a central role in the preservation of perishable food products. This innovative approach combines the distinct functional properties of 3–7 individual polymer layers into one single structure with enhanced protection, durability, and shelf life performance. Recently in 2025, Paszkiewicz et al. reported that PLA/PEF bi- or trilayer films show improved mechanical, thermal, and barrier properties, along with enhanced antimicrobial activity. In their study, the PEF layer was found to significantly contribute to both the antimicrobial performance and gas barrier characteristics.^152^ As per Kunamaneni, it is unlikely that PEF will completely replace PET in very near future given all the commercialization challenges around the PET infrastructure.^153^ However, the immediate value of PEF can be realized by utilizing it as a barrier coating material in biobased packaging products, such as those developed in the Paboco, the paper bottle project. Further studies of a similar nature are strongly recommended to deepen the understanding of PEF’s performance at the earliest market potential in food packaging applications. As with all new technology, it is important for companies to patent their inventions to provide legal protection to the novelty of their innovations. As of 2020, Avantium alone had its technology patented by 137 patent families.^146^ There are a wide range of patents for PEF covering copolymerization methods, chemical recycling, and applications including in the textile and food and beverage industry. These patents are owned by well-known names in the beverage industry including Coca Cola.^154^ Patents of PEF thermoforming and injection stretch blow molding could have applications for food and beverage containers or even trays and cups.^155–157^ There is also patented technology for highly viscous PEF for bottle manufacturing,^158^ laminated PEF films,^159^ orientated PEF films, and films containing a furandicarboxylate unit.^160,161^

### 5.5 Fibers and Textiles

PEF-based melt spun fibers have also been produced with promising mechanical properties. One of the challenges that remains with PEF based fibers is the elongation at break (<10%) which must be further improved to compete with PET yarn applications.^162^ Copolymers of PEF and polyethylene glycol (PEG) (i.e., PEGFs) were found to have a certain degree of degradability owing to the improved hydrophilicity of the copolyester. PEGF fibers were found to have uneven fiber distribution alongside broken wires and filaments. Further research is necessary to fabricate PEF or PEF copolyester fibers with certain spinnability and suitable mechanical properties.^163^ Due to the high price of FDCA in comparison to TA, it currently remains too expensive to produce PEF to compete with PET on an industrial scale. One approach to this issue that has been explored is to produce the copolyester, poly­(ethylene terephthalate-*co*-ethylene 2,5-furandicarboxylate) (PEFT). This involves partial substitution of the benzene moiety with the furan moiety. PEFT has been used to produce fibers through melt-spinning and hot-drawing processes in an industrially viable way. These fibers were found to have a comparable or even improved tenacity to PET fibers. They were also found to have a boiling water shrinkage quite similar to the PET fibers which demonstrated the fiber’s heat resistance.^164^ These results are encouraging for the use of PEFT in the fiber industry. PEF has also been successfully electrospun, creating fibers of different thicknesses, ranging from 180 nm to 2.13 μm, depending on the concentration of PEF and the solvent used.^165^ Studies have shown that the fiber formation conditions used for producing PET fibers are also suitable for PEF fibers, yielding mechanical properties appropriate for potential applications in the general semiengineering field.^166^ In a similar line to PET, Avantium in 2013 produced T-shirts from recycled PEF bottles.^167^ In the fiber industry, there are various patents available for the method of producing PEF yarn, by melt spinning with specific intrinsic viscosity and patents focused on PEF in tire manufacturing.^168–170^

### 5.6 3D Printing

Wang et al. successfully demonstrated a 3D-printed PEF-based climbing hook which was able to carry a load of up to 80 kg, showing excellent mechanical properties and load carry capacity. Other printed shapes including a Gyroid structure, a bionic flower, and a cube structure were tested, and it was found that they all showed excellent shape memory behavior by thermal stimulation. The successful 3D printing of PEF highlights its potential for applications in soft robotics, bone scaffolds, and biomimetic structures.^171^

### 5.7 Optoelectronic Devices

Alongside the opportunity for PEF in the packaging and textile industries, there is a diverse range of applications in other areas. Another area in which the polymer is being studied is for use in high-performance flexible optoelectronic devices. A flexible conductive film was successfully produced by combining PEF with silver nanowires. PEF is a suitable biobased option in this industry due to its excellent transparency of up to 90% and its enhanced adhesion to silver nanowires due to its furan moiety. The organic photovoltaic device achieved a power conversion efficiency of 6.7% which is promising for the replacement of PET with PEF in flexible electronic applications.^172^ Beyond the textile, food, and beverage industry, there is patented technology of PEF in other areas including functional mechanical parts and as a barrier layer in electrical cables, electrical conductors, or tubes.^173–175^

### 5.8 Biomedical Applications

Biocompatibility and antimicrobial properties are key characteristics of polymers that influence their use in biomedical applications, such as wound dressings, face masks, and protective clothing. These properties also expand their potential in nonmedical fields, including food packaging, water purification, and disinfection. In a cytotoxicity study using human keratinocytes, Svyntkivska et al. demonstrated that nonwoven electrospun PEF mats using 1,1,1,3,3,3-hexafluoro-2-propanol as the solvent are nontoxic to healthy cells. Moreover, the study found that incorporating silver particles on the fiber surface endowed the PEF mats with antimicrobial properties.^176^

### 5.9 Waste Water Treatment Drug Removal

The persistent presence of pharmaceutical residues in wastewater poses significant environmental and public health concerns, necessitating the development of advanced and sustainable removal strategies.^177^ In the work by Koltsakidou et al., biobased PEF/TiO_2_ nanocomposite films (up to 20 wt % TiO_2_) were developed via solvent evaporation for photocatalytic degradation of pharmaceutical pollutants.^178^ The composites exhibited a fine TiO_2_ dispersion, increased thermal stability, and unaffected crystallinity of the PEF matrix. Photodegradation tests targeting analgesic/anti-inflammatory mixtures showed that the 20% TiO_2_ formulation achieved 50% total organic carbon (TOC) reduction after 360 min. High-resolution mass spectrometry identified transformation products formed during the process. Importantly, the photocatalyst maintained stability and reusability across multiple cycles, outperforming conventional powdered TiO_2_ systems.

### 5.10 Limitations Related to Applications

While PEF offers these promising advantages, there are certain challenges that hinder its substitution for PET on an industrial scale. These include concerns over its low UV stability, intrinsic brittleness, higher production cost, and limited data on its recyclability and biodegradability under real-world conditions.^115^ Currently the cost per kilogram of producing PEF is between 8 and 10 times that of the cost of producing PET. These limitations have been addressed through various strategies, including copolymerization, nanocomposite fabrication, postprocessing treatments, optimization of PEF synthesis, and the development of recycling techniques aimed at aligning PEF with existing PET production and processing infrastructures. PEF is also being explored as a reinforcing or complementary component in biobased composites such as wood fibers to enhance the overall functional properties. One example of this is Carlsberg’s Fiber Bottle. Carlsberg launched a trial of its new Fiber Bottle in 2022. These bottles contained PEF polymer lining that protected the beer from the fiber outer shell, consisting of sustainably sourced wood fiber.^179^ Later in 2023, Zwicker et al. carried out a study to determine the willingness of consumers to purchase PEF bottles over PET bottles, where they included both a PEF plastic and a PEF paper bottle as well as a standard PET plastic bottle. It was found that 96.8% of people preferred the biobased bottles over the PET bottles, and of the biobased bottles, the PEF paper bottle was chosen more frequently as it looked more sustainable visibly, indicating that the public are looking for sustainable alternatives. This is very promising relating to cost as the PEF paper bottles require only a thin layer of PEF with a paper outer layer which can be separated from each other and both recycled separately along with producing a much higher volume of bottles as the cost of PEF is significantly lower for these bottles.^180^ However, more extensive and multidisciplinary research is essential to systematically address the current limitations of PEF to enable its adoption and integration into PET’s existing market infrastructure.

### 5.11 Life Cycle Analysis of PEF

Although PEF is often promoted as a more environmentally friendly alternative to PET, this claim needs further in-depth evaluation. Multiple LCA studies have been conducted to explore the environmental performance of PEF. In this section, we consolidate the most relevant and up-to-date LCA data on PEF and provide a critical analysis of its environmental impacts and associated challenges. Silverwood et al. recently published a review on multiple EoL options for PEF and its derivatives and provided recommendations for future PEF-based waste management strategies.^113^ In the case of PEF, most LCA studies are often referred to as cradle-to-grave assessments, which means evaluating the environmental impact of PEF throughout the entirety of its life. This includes, but is not limited to, identifying and quantifying the inputs and outputs throughout the PEF life cycle, assessing their potential environmental impacts, and exploring possible strategies for mitigating the environmental impacts associated with PEF. In the studies, the PEF production is associated with isolation of starting materials, conversion of the starting material to furanics to make the monomer, and the polymerization to PEF. Table 6 summarizes literature on LCA studies of PEF covering different system boundaries and impact assessment methods. The first comprehensive LCA of Avantium’s PEF was conducted by Eerhart et al. in 2012, in collaboration with the Nova-Institute, as part of the European PEFerence project.^27,181^ The LCA was performed within the initial scope of production technology associated with PEF and is compared with Ecoinvent data available for European plastics industry for bottle grade PET. The LCA includes the complete life stages of PEF, from the extraction or cultivation of raw materials through production, use, and disposal of the product, considering all relevant environmental aspects of FDCA and subsequent PEF production.^27^ The analysis of PEF was completed with the EoL being assumed to be the disposal of the PEF by the consumer to a solid waste incinerator without energy recovery (“grave”). To ensure a fair comparability with PET, the system boundaries were set to cradle-to-factory gate, which was later expanded to the cradle-to-grave framework according to ISO standards 14,040/44. The study indicates that the production of PEF can reduce the NREU approximately 40–50% while GHG emissions can be reduced approximately 45–55%, compared to PET.

**Table 6:** Literature on LCA Study of PEF with Various System Boundaries, Impact Assessment Methods and Key Findings Are Summarised

study	feedstock	system boundary	functional unit	allocation method	impact assessment method	GWP (kg CO_2_-eq/)	core findings
Eerhart et al., 2012 ^27^	corn based fructose for pef	cradle-to-grave	1 kg of PEF at factory gate	Mass-based	NA	-	Within the range of 45%–55% lower GHG emission, 40%–50% NREU than petroleum PET
Isola et al., 2017 ^182^	maize and potato	cradle-to-gate	1 g PEF	energy-based	ReCiPe 2008 midpoint	53.01 per g PEF	Use of maize was found to be effective in reducing the overall environmental impact compared to potatoes.
Jiang et al., 2020 ^67^	industrial carbon dioxide (CO_2_) and nonfood-derived biomass i.e. Xylan	cradle-to-factory gate	1 tonne PEF	mass and energy-based	CML2016 and Odor	3.0–3.7 per kg PEF	The GHG emission and energy consumption of this new route of PEF is higher than the conventional sugar-based PEF, while slightly lower than traditional petroleum-based PET.
Kim et al., 2022 ^20^	wheat straw (lignocellulosic feedstock)	cradle-to-gate	1 kg PEF	mass-based	TRACI 2.1	2.44–3.89 per kg PEF	Approximately, 134–163% GHG reduction compared to PET
Martikainen, Eero, 2022 ^183^	wood chip^b^	cradle-to-gate	1 kg of PEF	mass and energy-based	ReCiPe 2016 Midpoint	2.17 per kg PEF	PEF from a wood chip based biorefinery show lower environmental impacts compared to PET.
Stegmann et al., 2023 ^184^	starch from wheat, Bioethanol from sugar cane	cradle-to-grave	250 mL PEF bottle^c^	economic based, avoided burden approach for EoL.	IPCC 2013 GWP100a	2.4^a^per kg PEF	PEF offers 50–74% lower cradle-to-grave global warming potential (GWP) compared to PET after one recycling trip, mostly due to the nature of feedstock and material saving which is due to the superior gas barrier property and lightweight.

a The GWP results include the uptake of CO_2_ during the growth of the biobased feedstock and the emissions of biogenic CO_2_ when PEF is incinerated. Impact assessment after one recycling trip (as similar to PET).

b The process for PEF production from wood chip biorefinery.

c Injection blow molded monolayer bottle, providing minimum shelf life of at least 12 weeks for carbonated soft drinks.

Kim et al. studied the life cycle, GHG, water, and fossil-fuel consumption analysis of PEF including processes of feedstock farming, pretreatment, hydrolysis, conversion into furanics, recovery, polymerization into PEF, and on-site combined heat and power (CHP) generation.^20^ Jiang et al. studied the environmental impact of PEF produced from 99.5% pure industrial CO_2_ and a nonfood feedstock, namely, xylan at an industrial scale on the basis of data extended from lab-scale experimental studies, however limited by the database of software. The study covered CO_2_ emission, energy consumption, and production costs of PEF from the nonfood raw material.^67^ The nature of feedstock of this PEF would offer the advantage of not competing with the food sector, unlike the conventional PEF sourced from glucose and fructose. The study included the complete production pathway of PEF from xylan to furfural, and FDCA to PEF. The GHG emission and energy consumption of this new route of PEF is higher than the conventional sugar-based PEF, while still better than petroleum-based PET and other bioplastics like PLA. Eerhart et al. (2014) integrated the biorefinery concept into PEF production by coupling the organosolv process for carbohydrate extraction from lignocellulosic biomass with furan-based technology for converting carbohydrates into PEF.^185^ Their study presents comprehensive mass and energy balances for the production of PEF from lignocellulosic feedstocks, such as wheat straw. Also, the study highlights both the advantages and potential obstruction as well as assessing the degree of integration of this approach through modeling based on experimental data and extrapolating the findings to an industrial capacity. One of the major challenges that hinders the successful commercialization of second-generation biorefineries is the pretreatment methods of lignocellulosic biomass. Conventional pretreatment methods are often economically and environmentally undesirable due to their reliance on hazardous chemicals. In contrast, the organosolv technology presents a promising alternative, offering advantages in terms of both environmental compatibility and process efficiency.^186^ The integrated pathway for PEF production from lignocellulosic biomass demonstrates a high technical efficiency. However, to fully assess its viability, these results must be complemented by thorough economic and life cycle analysis. These evaluations are essential for determining the cost-effectiveness and attaining an understanding of the environmental impacts associated with the process. An analysis conducted by Martikainen et al. (2022) found that integrating the PEF production technology with a wood chip-based biorefinery can significantly reduce the environmental impact of PEF production. However, further research is required to optimize the process and integrate renewable energy sources in order to achieve additional reductions in environmental burdens.^183^

According to the Product Environmental Footprint guidelines, it is important to account for changes in carbon stocks over a defined period of time (e.g., 20 years) in both vegetation and soil due to Land Use Change (LUC) when conducting LCA studies of biobased polymers.^187^ LUC refers to the loss of carbon stored in the biosphere, which is a result from alterations in land use. In a seminal 2008 study, Searchinger et al. quantitatively calculated LUC associated with corn production and provided valuable insights into the release of carbon dioxide (CO_2_) from vegetation and soil during agricultural expansion.^188^ PEF is typically produced from biomass sources, such as corn starch or lignocellulosic materials, such as wheat straw. The cultivation of these feedstocks involves LUC, such as the conversion of natural ecosystems of land into agricultural land, which results in the release of sequestered carbon dioxide (CO_2_) from vegetation and soil. Incorporating LUC emission into life cycle assessment (LCA) studies of PEF in various system boundaries show a modest increase in greenhouse gas (GHG) emissions compared to assessments that exclude LUC impacts.^27^ Consequently, it is essential to evaluate lignocellulosic feedstocks as a potentially more sustainable biomass resource for the production of biobased polymers such as PEF. Isola et al. in 2017 performed LCA study for laboratory scale production of 1 g of PEF from potatoes and maize feedstocks.^182^ While a potato-based pathway exhibited a net negative environmental performance, incorporating polymer recycling into the production process significantly reduced the overall environmental impact of PEF. Also, the use of biobased EG, electricity export directly from the on-site CHP plant can further enhance the reduction of NREU, GHG, and GWP associated with PEF. Within a gate-to-grave system boundary, adopting alternative end-of-life pathways other disposal options such as chemical or mechanical recycling or the use of an incinerator with energy recovery options would likely lower the environmental impacts of PEF. Integrating renewable energy sources, energy supply directly from the CHP system will significantly reduce the GHG contribution of PEF.

### 5.12 Techno-Economic Analysis of PEF

TEA is a systematic approach for evaluating the technical and economic viability of products and processes in different stages of development and on various scales of production. For newly developed polymers like PEF, TEA plays an important role in understanding whether PEF can be produced competitively at an industrial scale. As a relatively new polymer, PEF suffers from limited publicly available data on its process efficiency, technological maturity, Technology Readiness Level (TRL), and operating costs. Moreover, the current production routes of PEF rely largely on nonhomogenous 1G and 2G biomass feedstocks. This makes the complete PEF production route much more complex compared to petrochemical-based PET. Considering the above factors, an overarching TEA is necessary to determine PEF’s entry to the market. This analysis must include the entire value chain for PEF production including the production and purification of starting material, i.e., HMF and FDCA.^189^ A formal TEA study on PEF was published by Louw et al., 2022 where they conducted an insightful TEA for the PEF production using different sources of biomass as feedstocks, including A-molasses (1G), sugar cane bagasse, and trash (2G), as well as a combination of the two 1G2G feedstocks. The study was integrated within biorefineries self-sufficient with bioenergy to evaluate the feasibility of sustainable production systems. For the 1G feedstock pathway, the existing boiler infrastructure of a conventional sugar mill was utilized to meet the energy demand. In contrast, due to the higher energy requirements associated with 2G and 1G2G processes, a newly implemented combined heat and power (CHP) plant was employed to ensure adequate energy supply and system efficiency.^190^ High purity and low-priced raw materials will determine industrial uptake and market demand for PEF. However, these specific topics fall outside the scope of this review and are therefore not discussed in detail here.

## 6 Current Industry Challenges and Recommendations

### 6.1 Current Industry Status of PEF

At this stage of this review, it has become evident for the readers that PEF is on the verge of becoming a key player in the commodity plastics sector, primarily due to its sustainable attributes and superior functional property compared to PET. According to recent analyses, the estimated market value of PEF in 2024 is USD 21.3 million.^191^ The same report forecasts that the global market value of PEF will reach approximately USD 47.0 million by 2034, reflecting a compound annual growth rate (CAGR) of 8.3% within the period from 2025 to 2034. However, the data on global market analysis of PEF differ from each other. For instance, one report forecasts a market demand of 0.3 thousand tonnes in 2025, which is expected to grow at an exceptional CAGR of 82%, reaching 122 thousand tonnes by 2035.^192^ In contrast, another report estimates that the global market value of PEF will reach USD 76.7 million by 2032.^193^ This variation in market research data of PEF may also be interpreted as an indication of its broad market potential upon successful resolution of current commercialization challenges, which are addressed herein.

Avantium’s commercial FDCA Flagship Plant YXY Technology in Delfzijl, The Netherlands has marked a significant milestone in initiating the commercial-scale production and expansion of PEF.^194^ A strategic collaboration and partnership across the entire value chain is essential to ensure a competitive market position for PEF. Sourcing of high-quality feedstocks at reasonable price, manufacturing the final product using the cost-effective technology, and establishing effective recycling processes are some of the critical components of the PEF value chain. Tereos is the feedstock partner of Avantium and provides High Fructose Syrup (HFS) for FDCA production.^195^ Selenis is the tolling partner for polymerization of PEF from FDCA. Beverage packaging currently represents the largest global market segment for PEF applications. Other emerging applications include cutlery products, liquid perfume bottles, textile yarns, cast films, as well as molded and extruded components. According to a 2022 market report, the global demand for beverage packaging is dominated by water (472.9 billion liters), followed by alcoholic beverages (272 billion liters), milk and dairy drinks (258.5 billion liters), and carbonated soft drinks (238.9 billion liters). To support these applications, regulatory approvals are essential. While US FDA has approved PEF as the food contact material, toward end of 2025, Avantium is expecting to commence the validation of compliance with the relevant food contact regulations in Europe. In terms of geographical location, Asia pacific region dominates the global PEF market demand, which is followed by Latin and North America, Europe, and Middle East & Africa.^196,197^ Avantium’s other strategic partners include Mitsui & Co. (Japan), Toyobo Co. Ltd. (Japan), ALPLA Group (Austria), Danone (France), Swicofil (Switzerland), Origin Materials (US), Eastman (US), AVA Biochem (Switzerland), Sulzer (Switzerland), Carlsberg (Denmark), Henkel (Germany), and Pangaia (Italy).^198^ The mentioned collaboration of globally established companies highlights the shared interest in realizing the commercial potential of PEF.

### 6.2 Delay in PEF Market Entry

The prolonged journey of PEF toward market entry since its initial discovery is largely due to strong competition from PET in terms of cost-effectiveness, scalability, and properties. Sustainability became a major focus over the past four decades only upon recognizing the finite nature of petrochemical feedstocks and their long-term environmental impact. Given the widespread presence of PET in everyday applications, it has become essential to identify a more sustainable alternative. Here, PEF has emerged as a promising candidate, and since 2010, research efforts on PEF have started gaining traction in response to growing environmental concerns. The key challenges contributing to the delayed market entry of PEF are presented in a simplified manner for the readers in Figure 10 and explained corresponding recommendations for addressing each issue.

**Figure 10:**
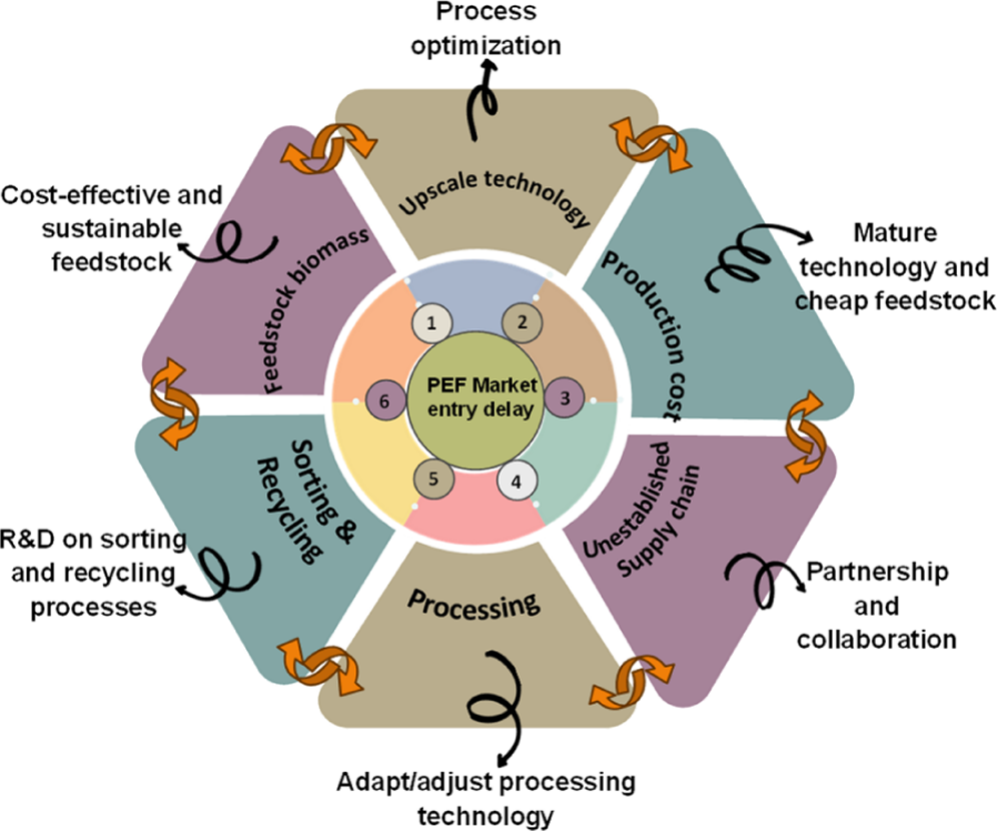
Infographic illustrating challenges and recommendations delaying PEF market entry.

#### 6.2.1 Challenges and Recommendations Associated with Industrial Upscaling

PEF can be synthesized through various processes, including melt polymerization via transesterification, solid-state post condensation polymerization, ring-opening polymerization, cyclodepolymerization of low-molecular-weight PEF, and enzymatic polymerization. The quality of the final PEF product is highly dependent on the selected polymerization route as its properties are extremely sensitive to reaction conditions. Melt polymerization via transesterification or SSP routes has been adopted for PEF production, owing to its similarity in the synthesis process with PET. Hence, upscaling of PEF production would have been benefited by utilizing the existing PET manufacturing infrastructure and commercially available equipment.^199^ However, PEF’s thermal sensitivity under high temperature and severe vacuum conditions along with the formation of byproducts often leads to inferior quality discolored PEF with impurities. Alternatively, production of PEF via “green” route like ROP addresses all the above-mentioned issues with product quality coupled with the added benefit of increased energy efficiency. The achievement in high-quality PEF production via ROP at the laboratory and pilot scales paves the way for its commercial feasibility.^81^

#### 6.2.2 Challenges and Recommendations Associated with Processing

The resultant high-molecular weight of PEF through the ROP technique increases the melt viscosity of PEF significantly compared to PET. This high viscosity is a double-edged sword: while it enhances mechanical strength and barrier properties, it simultaneously poses challenges for melt processing of PEF during the conversion into final products. Although PEF’s low melting temperature saves some energy, its narrow processing window of 210–220 °C, proximity to its degradation temperature, high viscosity, and shear sensitivity necessitate precise temperature control during processing.^42^ Fiber spinning, injection molding, blow molding, and oriented film stretching require high stretchability, which is significantly hindered by the high viscosity of PEF. In other words, substantial modifications to existing PET processing lines are necessary to accommodate the processing of PEF. Strategies like use of chain extenders, plasticizers, copolymerization, and specific catalysts to control molecular weight and the intrinsic viscosity can address these challenges.^200,201^

#### 6.2.3 Challenges and Recommendations Associated with Availability and Cost of Its Raw Materials (FDCA and EG)

The functional properties of PEF are comparable to or even superior to those of PET, making it a strong competitor in similar application areas. However, the distinctiveness of PEF lies in its fully biobased nature that aligns with global sustainability goals. In line with this, the sourcing of its basic monomer units, i.e., FDCA and EG from various biomass preferably 1G or 2G feedstocks, has been a significant challenge under current technological and economic conditions. FDCA is typically synthesized from HMF, which is primarily obtained from fructose and glucose derived from 1G feedstocks, such as corn and sugar cane. While 1G feedstocks offer higher efficiency in terms of both FDCA yield and product purity, their use raises concerns related to food security and land competition. In contrast, 2G feedstocks, such as lignocellulosic biomass, offer a sustainable and nonfood alternative. However, the current technologies for converting 2G biomass into HMF and subsequently FDCA often result in lower yields and inferior product quality. Furthermore, the current industrial process of FDCA production from 2G biomass is technologically immature and economically unviable for large-scale commercialization.

Global statistics indicate that the Asia-Pacific region and Latin & South America particularly Brazil and Argentina are emerging as the fastest-growing markets for PEF and FDCA.^197^ This growth is driven by increasing government regulations, environmental policy pressure, and heightened consumer awareness of sustainable packaging. However, there is a significant shortfall in commercial FDCA production despite its growing global demand.^202^ Currently, the largest operational FDCA production facility is Avantium’s YXY plant in The Netherlands, which became fully functional from 2024.^195^ However, its output is insufficient to meet worldwide FDCA demand and thus significantly impacts the raw material availability, solid supply chain infrastructure, and cost competitiveness in comparison to the raw materials of PET. In light of this, accelerated research and development efforts are needed to advance the FDCA production technology that offer improved efficiency and are suitable for industrial scale-up while maintaining high product quality. In addition, government support by subsidizing and incentivizing tax of such “green” chemicals strengthen the value chain for PEF production and help meet the growing global market demand.^203^

#### 6.2.4 Challenges and Recommendations Associated with Recycling

Efficient recycling of polymers, which is the key to ensuring the quality of the final recycled product, requires advanced separation techniques to isolate individual types with high precision. In the case of PET, the mechanical recycling stream can tolerate a maximum of only 2–5% PEF contamination to maintain high product quality.^31,32^ PEF shares many technical advantages with PET such as similar appearance and physicochemical properties, largely due to their comparable chemical structures. While this similarity allows PEF to serve as a viable replacement for PET in various applications, it also poses a significant challenge during recycling, particularly at the sorting stage, where distinguishing between the two becomes difficult. Instead of relying on traditional manual or density-based sorting methods, the near-infrared (NIR) sorting technology has proven to be effective in distinguishing PEF from PET. This highlights the need for all plastic sorting facilities to be equipped with relatively expensive NIR systems.^204^ Additionally, measures such as smart labeling like incorporating QR codes, hallmarks, fluorescent labels on PEF products can further enhance the accuracy of sorting, especially when separating PEF from other plastics with densities lower than water. However, such technological and logistical upgrades in sorting facilities will add up to the overall cost of recycling PEF. Like PET, the mechanical recycling of PEF is limited by the gradual product quality deterioration with each recycling cycle. Chemical recycling, therefore, plays a crucial role in closing the loop for PEF without downcycling its properties, and achieves new high-quality PEF.^136^ In chemical recycling, the depolymerization of PEF produces furan-based macrocycles, which can subsequently be repolymerized into high-quality PEF.^81,137^ According to the literature, it is highly recommended to avoid alcoholysis and hydrolysis of PEF streams contaminated with PET or other biobased plastics as these processes lead to the formation of unwanted byproducts that are difficult to separate. In contrast, glycolysis has proven to be effective in addressing contamination issues, making it the most favorable process at both laboratory and industrial scales.^135^ Number of papers published to date is insufficient to draw a clear picture on PEF chemical recycling as most studies have been limited to the laboratory scale. Therefore, further pilot-scale research is necessary to validate the efficacy of the process for industrial-scale applications.

## 7 Conclusions

Poly­(ethylene furanoate) (PEF) is a 100% biobased polyester primarily derived from 2,5-furandicarboxylic acid (FDCA) and ethylene glycol (EG). Latest research suggests that PEF has emerged as one of the most promising sustainable alternatives to poly­(ethylene terephthalate) (PET). This review has examined the complete and up-to-date literature on PEF across a wide breadth of research areas including synthesis strategies, structure–property relationships, processing behavior, performance characteristics, end-of-life pathways, life cycle assessments, as well as sorting and recycling prospects in various setups. It critically evaluates the chronological progress of PEF research in these areas to provide a clear understanding and map the developments systematically through tabulated summaries. The existing research literature demonstrates that PEF exhibits most of the desirable qualities of PET such as high mechanical strength, thermal stability, and processability, while also offering additional advantages of superior gas barrier properties, enzymatic degradability, and lower carbon emissions across its life cycle. These are the attributes that positions PEF as a strong candidate for packaging, textiles, and a range of other applications where PET currently dominates. Moreover, latest studies indicate that PEF can be processed within PET’s existing industrial-scale production and recycling infrastructures, thus providing further advantage for its potential integration into existing markets. Despite these advances, several critical gaps remain and require further attention. This review highlights unresolved challenges across technoscientific research and commercial aspects of PEF and provides directions for future investigations that will be crucial for advancing its development and industrial adoption. The literature on PEF synthesis is rich in laboratory-scale studies based on the polycondensation pathway, which is highly energy-intensive, associated with significant carbon emissions, and often yields low-molecular weight, discolored PEF due to byproduct formation. As a result, this route has limitations for industrial translation and is considered less environmentally sustainable. ROP is regarded as a more environmentally sustainable technique for PEF synthesis and has shown considerable potential for producing high-molecular-weight PEF. However, further research is needed to gain a deeper understanding of its complexities and limitations, particularly with respect to its scalability and industrial adoption. While life cycle assessments consistently highlight the significantly lower carbon footprint of PEF compared to fossil-based PET, uncertainties regarding land use change (LUC), feedstock type (1G, 2G or mix), and its availability still exist and require further investigation. Besides ensuring high-purity, high-molecular-weight PEF via ROP, assessing EoL strategies is essential for a comprehensive understanding of PEF’s sustainability. Literature suggests that PEF is compatible with existing PET mechanical recycling systems, with the need for minor adjustments in parameters enabling its processing within current PET recycling infrastructure. An additional advantage is both PEF and PET can be separated from each other using NIR technology, which is advantageous for a mixed plastics stream. Chemical recycling approaches for PEF are also advancing while considering its structural similarity to PET. However, the biodegradability of PEF is inconclusive, with limited understanding of its long-term degradation under industrial and natural conditions. Also, the ecological impact of its degradation products is not fully understood, creating a void of knowledge and indicating the need for further investigation. Application of standardized biodegradation methodologies for assessing PEF’s biodegradability would improve comparability across various studies. However, enzymatic recycling offers a promising sustainable pathway, but process efficiencies vary across different enzymes and environmental conditions, which highlights the need for further research. Additionally, integrating the supply chain and engaging various stakeholders, along with active industrial investment in interdisciplinary research and scale-up studies, are essential to translate laboratory-scale advancements of PEF into commercially viable industrial processes. This perfectly aligns with efforts to support the broader adoption of PEF as a biobased alternative of PET. Hence, PEF’s development can be realized by interconnected efforts across its synthesis, processing, degradation, recycling, and environmental policy enforcement.
